# Serotonin transporter-mediated molecular axis regulates regional retinal ganglion cell vulnerability and axon regeneration after nerve injury

**DOI:** 10.1371/journal.pgen.1009885

**Published:** 2021-11-04

**Authors:** Rody Kingston, Dwarkesh Amin, Sneha Misra, Jeffrey M. Gross, Takaaki Kuwajima

**Affiliations:** 1 Department of Ophthalmology, The Louis J. Fox Center for Vision Restoration, Pittsburgh, Pennsylvania, United States of America; 2 Department of Developmental Biology, The McGowan Institute for Regenerative Medicine, The University of Pittsburgh School of Medicine, Pittsburgh, Pennsylvania, United States of America; University of Iowa, UNITED STATES

## Abstract

Molecular insights into the selective vulnerability of retinal ganglion cells (RGCs) in optic neuropathies and after ocular trauma can lead to the development of novel therapeutic strategies aimed at preserving RGCs. However, little is known about what molecular contexts determine RGC susceptibility. In this study, we show the molecular mechanisms underlying the regional differential vulnerability of RGCs after optic nerve injury. We identified RGCs in the mouse peripheral ventrotemporal (VT) retina as the earliest population of RGCs susceptible to optic nerve injury. Mechanistically, the serotonin transporter (SERT) is upregulated on VT axons after injury. Utilizing SERT-deficient mice, loss of SERT attenuated VT RGC death and led to robust retinal axon regeneration. Integrin β3, a factor mediating SERT-induced functions in other systems, is also upregulated in RGCs and axons after injury, and loss of integrin β3 led to VT RGC protection and axon regeneration. Finally, RNA sequencing analyses revealed that loss of SERT significantly altered molecular signatures in the VT retina after optic nerve injury, including expression of the transmembrane protein, *Gpnmb*. GPNMB is rapidly downregulated in wild-type, but not SERT- or integrin β3-deficient VT RGCs after injury, and maintaining expression of GPNMB in RGCs via AAV2 viruses even after injury promoted VT RGC survival and axon regeneration. Taken together, our findings demonstrate that the SERT-integrin β3-GPNMB molecular axis mediates selective RGC vulnerability and axon regeneration after optic nerve injury.

## Author summary

Retinal ganglion cells (RGCs) are the only neurons in the retina that convey visual information to the brain, doing so via the optic nerve. Major causes of optic nerve damage and subsequent RGC death are ocular trauma and optic neuropathies. At the end-stage pathology in the retina, remaining RGCs have high neuroprotective capacity, and active resilience mechanisms exist. In contrast, in this study, we demonstrate neurodegenerative mechanisms based on the early susceptibility of peripheral ventrotemporal (VT) RGCs to degeneration after optic nerve injury. We identified the serotonin transporter (SERT) as the factor mediating VT RGC vulnerability and axon regeneration. Loss of SERT affected activation of integrin β3 and gene expression in the peripheral VT retina, leading to peripheral VT RGC protection and axon regeneration. Interestingly, GPNMB, a newly identified molecule mediating neuroprotection and axon regeneration in this study, is expressed in all retinal regions. However, GPNMB expression is acutely downregulated by the presence of SERT in the peripheral VT retina, leading to regional differential vulnerability of RGCs. These findings provide new molecular mechanisms underlying selective RGC vulnerability and clues that could inform further neuroprotective and regenerative strategies.

## Introduction

Mature retinal ganglion cell (RGC) axons that comprise the optic nerve degenerate, RGCs die and blindness ensues after ocular trauma or during optic neuropathies such as glaucoma. Ocular trauma, such as facial fractures, leads to 67% of patients who have ocular injuries, of which 4% present with optic nerve abnormalities [1]. Retinal nerve fiber reduction and RGC loss after ocular trauma occur independently of age and sex [2]. Following injury, RGC axons degenerate, and RGCs eventually die rapidly or chronically depending on the causes and magnitude of the damage [2]. Human RGCs also lack any regenerative capacity and they do not recover from damage. However, there are no treatments or FDA-approved drugs that maintain the optic nerve and RGCs or that promote axon regeneration after ocular trauma or during optic neuropathies.

A number of studies have described intrinsic and extrinsic molecules and signaling pathways that mediate RGC death or survival pathways as well as axon regeneration after optic nerve crush (ONC) [3–9]. Targeting these molecules and pathways holds tremendous potential for establishing therapeutic interventions after optic trauma. However, the magnitude of vulnerability and onset of RGC death after injury or in the diseased retina varies depending on the RGC subtypes that are affected [10–15]. 46 RGC types in adult mouse retina display differential RGC vulnerability and resilience with distinct molecular signatures, some of which have been identified as novel neuroprotection and/or axon regeneration-related genes [16]. Adding complexity to various RGC types, the reduction of retinal axon layer thickness and loss of RGCs does not occur evenly in the whole retina but is instead localized to specific retinal regions at the early stages of neurodegeneration in optic neuropathies and after ocular trauma [17–20]. However, little is known about whether the same molecular mechanisms mediate neurodegeneration in all retinal regions or whether region-specific pathways exist to influence the rate or magnitude of degeneration in select regions, leaving others unaffected. Thus, a better understanding of the phenomenon of selective vulnerability and identification of the molecular mechanisms underlying this region-specificity is required for the development of targeted therapeutic approaches that protect those susceptible RGCs.

Axonal connections from RGCs in specific retinal regions are required for the formation of visual maps [21]. In mice, RGCs in all regions except the peripheral ventrotemporal (VT) retina project contralaterally. We and others found that several axon guidance and transcription mechanisms mediate the contralateral retinal axon projection during development [22–25]. In contrast, axons from the peripheral VT RGCs project ipsilaterally. The regulatory mechanisms of ipsilateral RGC specification, axon projection and targeting into the brain are distinguished from the mechanisms of contralateral RGC development [21]. The serotonin transporter (SERT, encoded by *Slc6a4*), is highly expressed in VT RGCs during retinal development and at early postnatal stages [26–28]. SERT regulates activity-dependent refinement of ipsilateral retinal axons and eye-specific segregation in thalamic and midbrain targets during postnatal stages [28,29]. However, the expression and functions of SERT in the adult retina after ocular trauma remain incompletely understood.

Utilizing a well-established optic nerve crush (ONC) mouse model, first we characterized regional differential RGC vulnerability at the early time points of axon degeneration. We demonstrate that the peripheral VT retina in mouse, the region where ipsilaterally-projecting RGCs are located, is more susceptible to degeneration after ONC compared to other retinal regions. To understand the molecular mechanisms underlying this susceptibility, we focused on SERT, which is upregulated on VT axons after ONC, and demonstrate that it functions in peripheral VT RGC death and impaired axon regeneration. Furthermore, loss of SERT in the peripheral VT retina attenuates the activation of integrin β3 and affects molecular signatures in the peripheral VT retina including *Gpnmb*, which results in reduced VT RGC death and enhanced axonal regeneration. Taken together, this study identifies a novel molecular axis underlying selective regional vulnerability of RGCs after ocular trauma.

## Results

### Peripheral VT RGCs are more susceptible to degeneration compared to other RGCs

**Fig 1:**
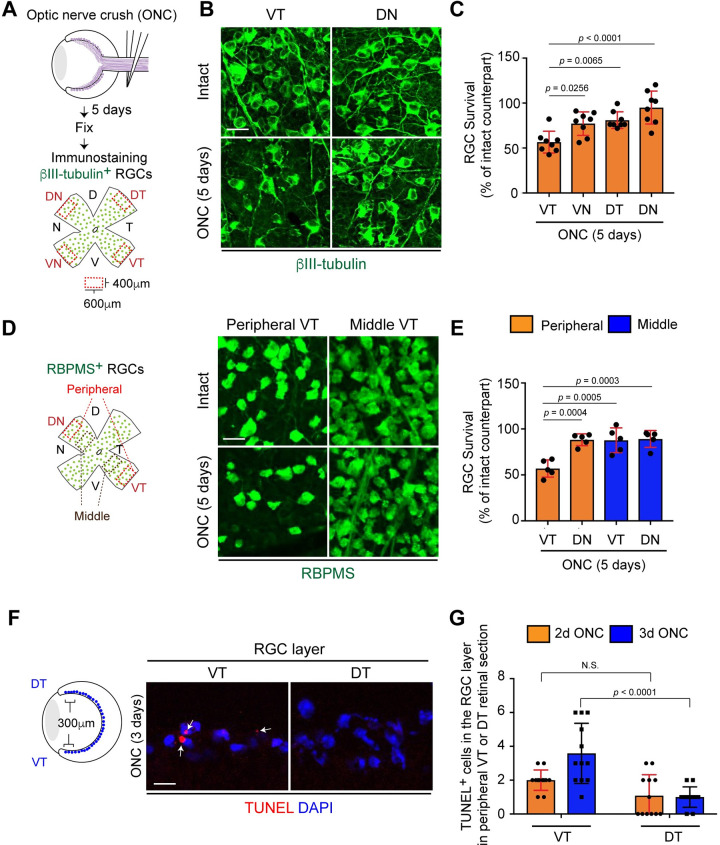
Peripheral VT RGCs are more vulnerable to optic nerve injury than other RGCs. (A) Schema of βIII-tubulin^+^ RGC survival analysis in four quadrants of the retina five days after ONC. (B-C) βIII-tubulin^+^ peripheral VT RGCs are more vulnerable to injury compared to other RGCs in the peripheral retina after ONC (n = 8 mice, one-way ANOVA). (D-E) RBPMS^+^ RGCs in the peripheral VT retina are more vulnerable than RGCs in the middle VT, DN or the peripheral DN retina five days after ONC (n = 5 mice, one-way ANOVA). (F-G) The larger number of TUNEL^+^ RGCs in the peripheral VT retina per cryosection three days after ONC is detected compared to DT retina (n = 12 sections from 3 mice/condition, two-way ANOVA). Data presented as mean ± SD. N.S., not significant; Scale bars represent 20 μm.

RGC loss is observed within five days after optic nerve crush (ONC) [30]. Thus, we first examined whether RGC vulnerability varies among different RGC locations at the early stages of axon degeneration, five days after ONC in C57BL/6J mice (hereafter referred to as WT mice). We quantified the number of RGCs using a pan-RGC marker, βIII-tubulin, in four quadrants of the peripheral retina (400 μm from the peripheral edge, 600 μm width): ventrotemporal (VT), ventronasal (VN), dorsotemporal (DT) and dorsonasal (DN) in 8-week old wild-type (WT) mice (Fig 1A). After whole-mount retinal immunostaining, we compared the number of RGCs in four quadrants of the injured retina from the left eye and intact contralateral retina from the right eye in the same animal to generate the RGC survival rate in each quadrant of the peripheral retina. We found that 95.0% of DN RGCs, 81.0% of DT RGCs and 77.2% of VN RGCs survived, while only 56.6% of VT RGCs survived (Fig 1B and 1C). Next, we reevaluated vulnerability of peripheral VT RGCs compared to DN RGCs with another RGC marker, the RNA-binding protein RBPMS. We then examined VT RGC survival rate in the middle region between the peripheral edge and optic disc (Fig 1D and 1E). We also found that only 57% of peripheral VT RGCs survived while >87% of peripheral DN, middle VT and DN RGCs survived (Fig 1E). To confirm whether an acute reduction in the number of VT RGCs after ONC is caused by cell death, we performed a TUNEL assay (Fig 1F and 1G). A significant increase in TUNEL^+^ cells in the VT RGC layer was detected three days after ONC when compared to the DT retina (Fig 1G). Next, we tested the hypothesis that resistant RGC subtypes such as αRGCs that express osteopontin (OPN) [13] may not be expressed in the peripheral VT retina. However, OPN^+^ αRGCs were evenly located in four quadrants of the peripheral retina (S1A and S1B Fig). Thus, peripheral VT RGCs are more vulnerable to injury than RGCs in other regions.

### VT RGCs die after elevation of SERT expression on VT axons following optic nerve injury

**Fig 2:**
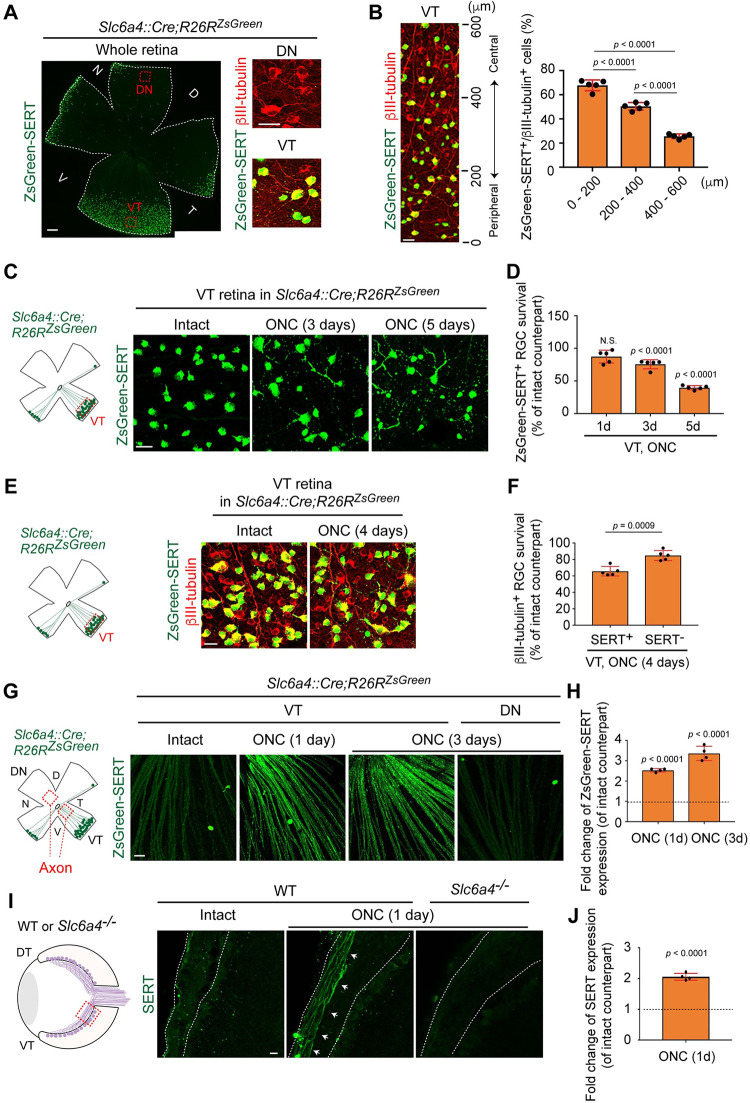
VT RGC death occurs after SERT expression on VT axons is elevated following optic nerve injury. (A) Ipsilateral RGCs (ZsGreen-SERT^+^ RGCs) are located in the peripheral VT, but not DN retina of *Slc6a4*::*Cre;R26R*^*ZsGreen*^ mice. (B) Ipsilateral RGCs (ZsGreen-SERT^+^ RGCs) are more predominantly located in the peripheral (0–200 or 200–400 μm) compared to the central (400–600 μm) VT retina in *Slc6a4*::*Cre;R26R*^*ZsGreen*^ mice (n = 5 mice, one-way ANOVA). (C-D) Time-course of ipsilateral RGC (ZsGreen-SERT^+^ cells) death in the peripheral VT retina after ONC (n = 5 mice/condition, one-way ANOVA). (E) In the peripheral VT retina four days after ONC in *Slc6a4*::*Cre;R26R*^*ZsGreen*^ mice, ipsilaterally-projecting RGCs (ZsGreen-SERT^+^/βIII-tubulin^+^) are more vulnerable to injury than contralaterally-projecting RGCs (ZsGreen-SERT-negative/βIII-tubulin^+^). (F) Quantitative analysis of ipsilateral (ZsGreen-SERT^+^/βIII-tubulin^+^) and contralateral (ZsGreen-SERT-negative/βIII-tubulin^+^) RGC survival (%) in the peripheral VT retina four days after ONC (n = 5 mice, two-tailed unpaired t-test). (G-H) In *Slc6a4*::*Cre;R26R*^*ZsGreen*^ mice, ZsGreen-SERT expression (*Slc6a4*::*Cre-*derived ZsGreen expression) is upregulated on VT axons (Axon) one and three days after ONC (n = 4 mice/condition, one-way ANOVA). (I) Endogenous SERT expression is upregulated on injured VT axons of WT mice one day after ONC (arrows) compared to the intact VT axons. SERT expression is not observed on injured VT axons of *Slc6a4*^*-/-*^ mice. (J) Quantitative analysis of SERT expression changes in axons of VT retina one day after ONC compared to the intact counterparts (n = 4 mice, two-tailed unpaired t-test). Data presented as mean ± SD. N.S., not significant; Scale bars represent 20 μm (A (high magnifications), B, C, E, G, I) and 200 μm (A (whole retina)).

The peripheral VT mouse retina has unique characteristics of RGCs: most peripheral VT RGCs are ipsilaterally-projecting RGCs [21]. To characterize ipsilateral RGC death in the peripheral VT retina after ONC, we used the genetic tool, *Slc6a4*::*Cre;R26R*^*ZsGreen*^ mouse (serotonin transporter (SERT), encoded by *Slc6a4*), and visualized ipsilateral RGCs showing ZsGreen-SERT expression as we previously utilized [26] (Fig 2A). We confirmed localization of ipsilateral RGCs based on ZsGreen-SERT expression in VT RGCs, but not DN RGCs in *Slc6a4*::*Cre;R26R*^*ZsGreen*^ mice (Fig 2A). We then examined the distribution of ipsilateral RGCs (ZsGreen-SERT^+^/βIII-tubulin^+^) from the peripheral edge (0 μm) to the central region (600 μm) in the uninjured adult VT retina (Fig 2B). 67.8%, 50.4% or 25.6% of RGCs in 0–200, 200–400 or 400–600 μm from the peripheral edge in the VT retina, respectively express ZsGreen-SERT. Thus, ipsilateral RGCs are more peripherally located in the VT retina.

We then examined the time course of ipsilateral RGC death after ONC in *Slc6a4*::*Cre;R26R*^*ZsGreen*^ mice, and found that ipsilateral RGCs (ZsGreen-SERT^+^ cells) began to die three days after ONC and only 39.6% of those RGCs survived five days after ONC (Fig 2C and 2D). In fact, the peripheral mouse VT retina contains not only ipsilaterally-projecting RGCs but also small populations of late-born contralaterally-projecting RGCs [22]. We then compared vulnerabilities of ipsilaterally-projecting RGCs (ZsGreen-SERT^+^/βIII-tubulin^+^) and contralaterally-projecting RGCs (ZsGreen-SERT-negative/βIII-tubulin^+^) within the peripheral VT retina four days after ONC (Fig 2E and 2F). We found that ipsilaterally-projecting RGCs are more vulnerable to injury than contralaterally-projecting RGCs within the peripheral VT retina (Fig 2F). Thus, ipsilaterally-projecting RGCs in the peripheral VT retina are the most susceptible to injury.

During the analysis of VT RGC death in *Slc6a4*::*Cre;R26R*^*ZsGreen*^ mice, we found that ZsGreen-SERT (*Slc6a4*::*Cre*-derived ZsGreen) expression was upregulated on VT RGC axons within one day after ONC, and its expression remained high three days after ONC (Fig 2G and 2H). However, its elevated expression was not observed on DN RGC axons after ONC (Fig 2G). For further analysis of altered SERT expression on VT axons after ONC, we utilized SERT antibody to examine endogenous SERT expression changes in response to degeneration in WT mice (Fig 2I and 2J). In the intact VT axons, SERT expression was extremely low while SERT was significantly upregulated on VT axons one day after ONC (2.06-fold change) (Fig 2J). We also confirmed that SERT expression was undetected in the injured VT axons of SERT-deficient, *Slc6a4*^*-/-*^ mice [31] (Fig 2I). Thus, SERT expression on VT RGC axons is elevated in response to injury, and this could contribute to ipsilateral RGC death in the VT retina.

### Loss of SERT leads to neuroprotection of VT RGCs after injury

**Fig 3:**
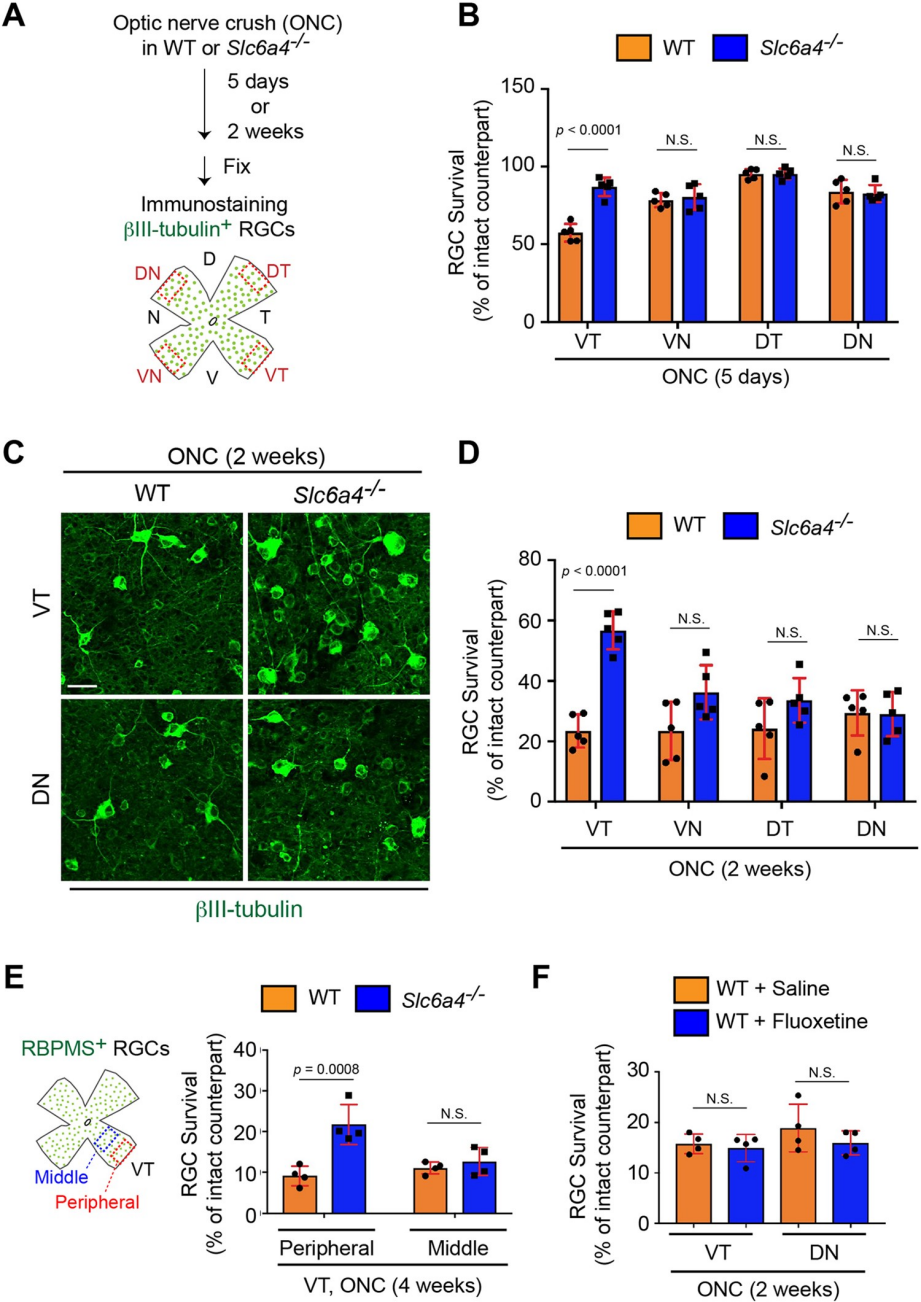
Loss of SERT leads to neuroprotection of peripheral VT RGCs after optic nerve injury. (A) Schema of RGC survival analysis in the retina five days or two weeks after ONC in WT and *Slc6a4*^*-/-*^ mice. (B) Five days after ONC, WT VT RGCs are markedly reduced, while ~80% of VT RGCs in *Slc6a4*^*-/-*^ mice still survive (n = 5 mice/condition, two-way ANOVA). (C-D) Two weeks after ONC, VT RGCs in *Slc6a4*^*-/-*^ mice are more resistant to injury compared to RGCs in other regions in *Slc6a4*^*-/-*^ mice or all regions in WT mice (n = 5 mice/condition, two-way ANOVA). (E) Four weeks after ONC, peripheral, but not middle VT RGCs in *Slc6a4*^*-/-*^ mice are more protective compared to those in WT mice (n = 4 mice/condition, two-way ANOVA). (F) Fluoxetine, a serotonin reuptake inhibitor has no neuroprotective effects in VT and DN RGCs two weeks after ONC (n = 4 mice/condition, two-way ANOVA). Data presented as mean ± SD. N.S., not significant; Scale bar represents 20 μm.

To test the hypothesis that SERT mediates RGC vulnerability in the peripheral VT retina after ONC, we investigated RGC survival at two time points, five days (early RGC degeneration) and two weeks (late RGC degeneration) after ONC in *Slc6a4*^*-/-*^ constitutive mutant mice [31] (Fig 3A). We found that the number of RGCs in each quadrant of the peripheral contralateral intact retina in *Slc6a4*^*-/-*^ mice was similar to that in WT (S2 Fig). Five days after ONC, in the VT retina, only 57.4% of RGCs in WT mice survived, while 87.0% of RGCs in *Slc6a4*^*-/-*^ mutant mice survived (Fig 3B). In other regions of the retina, WT and *Slc6a4*^*-/-*^ RGCs survived at the same extent (Fig 3B). Two weeks after ONC, in the VT retina, only 23.4% of VT RGCs in WT mice survived, while 56.8% of VT RGCs in *Slc6a4*^*-/-*^ mutant mice survived (Fig 3C and 3D). In contrast, in other regions, WT and *Slc6a4*^*-/-*^ mutant mice showed similar low RGC survival rates (Fig 3D). Next, we investigated the long-term neuroprotective effects in the peripheral VT retina of *Slc6a4*^*-/-*^ mutant mice after ONC (Fig 3E). In the peripheral VT retina four weeks after ONC, 21.7% of RGCs in *Slc6a4*^*-/-*^ mutant mice survived while only 9.2% of RGCs in WT mice survived. However, in the middle region of the VT retina where endogenous SERT is absent, WT and *Slc6a4*^*-/-*^ RGCs were similarly vulnerable to injury (Fig 3E). Loss of SERT alters serotonergic signaling and influences serotonin hemostasis [28,32,33]. Thus, to test the hypothesis that VT RGC protection after ONC in *Slc6a4*^*-/-*^ mutant mice could be caused by the inhibition of serotonin transport, fluoxetine, an inhibitor of serotonin reuptake was prepared fresh daily and given by intraperitoneal injection one day before ONC and daily after ONC for two weeks (Fig 3F). We found no significant neuroprotective effects of fluoxetine in VT and DN retina two weeks after ONC (Fig 3F). However, fluoxetine treatments in WT mice resulted in a reduced number of microglia/macrophages at the lesion site when compared to control, saline in WT, while *Slc6a4*^*-/-*^ mutants and WT mice showed a similar number of these immune cells (S3 Fig). Thus, SERT mediates the death of RGCs in the peripheral VT retina after ONC, while fluoxetine affects the whole retinal environment.

### Integrin β3 mediates VT RGC death after injury

**Fig 4:**
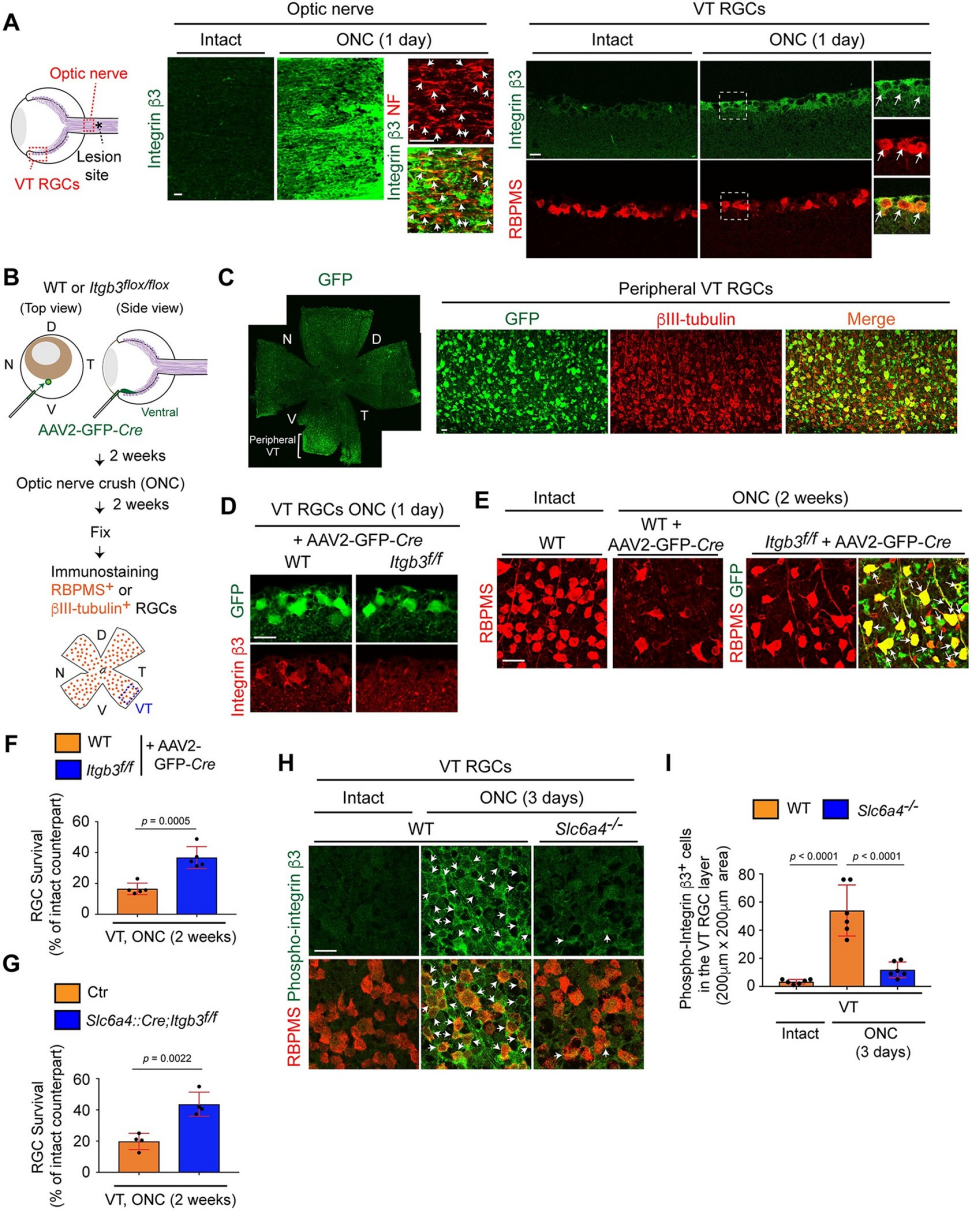
Elevated expression of integrin β3 following injury leads to VT RGC death. (A) One day after ONC, integrin β3 is upregulated in neurofilament (NF) ^+^ retinal axons (arrows) and RBPMS^+^ RGCs in the VT retina (arrows), but not in the intact axons and RGCs. (B) Schema of deletion of *Itgb3* after intravitreal injection of AAV2-GFP-*Cre* virus into the peripheral ventral retina of *Itgb3*^*flox/flox*^ mice and RGC survival analysis in the peripheral VT RGCs two weeks after ONC. (C) Most peripheral VT βIII-tubulin^+^ RGCs express GFP. (D) Integrin β3 expression in the VT retina of *Itgb3*^*flox/flox*^ mice + AAV2-GFP-*Cre* virus was eliminated one day after ONC. (E-F) Peripheral VT RGCs of the *Itgb3* ^*flox/flox*^ retina + AAV2-GFP-*Cre* virus are protected compared to WT retina two weeks after ONC and those RGCs express GFP-Cre (arrows) (n = 5 mice/condition, two-tailed unpaired t-test). (G) RGCs in the peripheral VT retina of *Slc6a4*::*Cre;Itgb3*^*flox/flox*^ mice are more resistant to injury than control (Ctr) two weeks after ONC (n = 4 mice/condition, two-tailed unpaired t-test). (H-I) Phosphorylated integrin β3^+^ RGCs are observed in the VT retina of WT mice, but not *Slc6a4*^*-/-*^ mice three days after ONC or in the intact retina (arrows). The number of phosphorylated integrin β3^+^ in the injured and intact VT retina of WT or in the injured VT retina of *Slc6a4*^*-/-*^ mice three days after ONC was counted (n = 6 mice/condition, one-way ANOVA). Data presented as mean ± SD. N.S., not significant; Scale bars represent 20 μm.

To explore the molecular mechanisms of SERT-mediated VT RGC death after ONC, we focused on integrin β3, which binds to SERT and regulates SERT-induced functions in platelets and the nervous system [33,34]. First, we examined expression of integrin β3 in the intact and injured optic nerve and RGCs (Fig 4A). We found that integrin β3 was upregulated in the injured optic nerve one day after ONC compared to the intact nerve (2.01 ± 0.43-fold change, n = 4 mice, two-tailed unpaired t-test, P = 0.0033). Moreover, 90.7% of injured VT RGCs expressed integrin β3, while only 3.5% of intact VT RGCs expressed integrin β3 (n = 4 mice/86 intact and 90 injured RGCs, two-tailed unpaired t-test, P<0.0001) (Fig 4A).

We next investigated the effects of integrin β3 on VT RGC vulnerability after ONC. To delete *Itgb3* in most RGCs of the peripheral VT retina, AAV2-GFP-*Cre* virus was intravitreally injected into the peripheral ventral retina of WT or of *Itgb3*^*flox/flox*^ mice (Fig 4B and 4C). Two weeks after virus infection, ONC was performed. The RGC survival rate in peripheral VT RGCs of *Itgb3* ^*flox/flox*^ retina expressing AAV2-GFP-*Cre* (referred to as *Itgb3*^*-/-*^ retina) was compared to WT retina expressing AAV2-GFP-*Cre* (referred to as WT retina). First, we confirmed that GFP-Cre expression was detected in ~95% of RGCs in the peripheral VT retina (Fig 4C). Elevated integrin β3 expression after ONC was eliminated in the *Itgb3*^*-/-*^ VT retina (Fig 4D). Two weeks after ONC, in the peripheral VT retina, 36.8% of RGCs of the *Itgb3*^*-/-*^ retina survived and those RGCs express GFP-Cre, while only 16.5% of RGCs of WT retina survived (Fig 4E and 4F). To confirm effects of integrin β3 on ipsilateral (SERT^+^) VT RGC death after ONC, *Slc6a4*::*Cre;Itgb3*^*flox/flox*^ mice were generated (Fig 4G). In the VT retina, 43.6% of *Itgb3*^*-/-*^ RGCs survived, while only 19.8% of control RGCs (*Slc6a4*::*Cre;Itgb3*^*+/+*^) survived. In fact, integrin β3 was also upregulated in dorsal RGCs after ONC where SERT is not expressed (S4A Fig). We next examined the regional specificity of integrin β3 functions in RGC death. To delete *Itgb3* in most RGCs of the peripheral dorsal retina, AAV2-GFP-*Cre* virus was intravitreally injected into the peripheral dorsal retina of WT or of *Itgb3*^*flox/flox*^ mice, and we investigated RGC survival in *Itgb3*^*-/-*^ RGCs of the dorsal retina (S4B Fig). We found that dorsal (e.g. DN) RGCs of the *Itgb3*^*-/-*^ retina were not protected after ONC (S4C and S4D Fig).

Since both SERT and integrin β3 mediate VT RGC death after ONC, we then tested the hypothesis that SERT mediates activation of integrin β3 after ONC. Thus, we examined the number of phosphorylated integrin β3^+^ RGCs in the VT retina after ONC in the VT retina of WT and *Slc6a4*^*-/-*^ mutant mice three days after ONC when WT VT RGCs begin to die (Fig 4H and 4I). The larger number of phosphorylated integrin β3^+^ RGCs was detected in the VT retina of WT mice three days after ONC (82.0%) compared to the intact retina (3.58%) (Fig 4I). However, even three days after ONC, only 12.2% of RGCs in *Slc6a4*^*-/-*^ mutant mice expressed phosphorylated integrin β3 (Fig 4I). Thus, SERT-mediated integrin β3 phosphorylation leads to RGC death after ONC in the VT retina.

### Retinal axon regeneration is promoted by loss of SERT and Integrin β3

**Fig 5:**
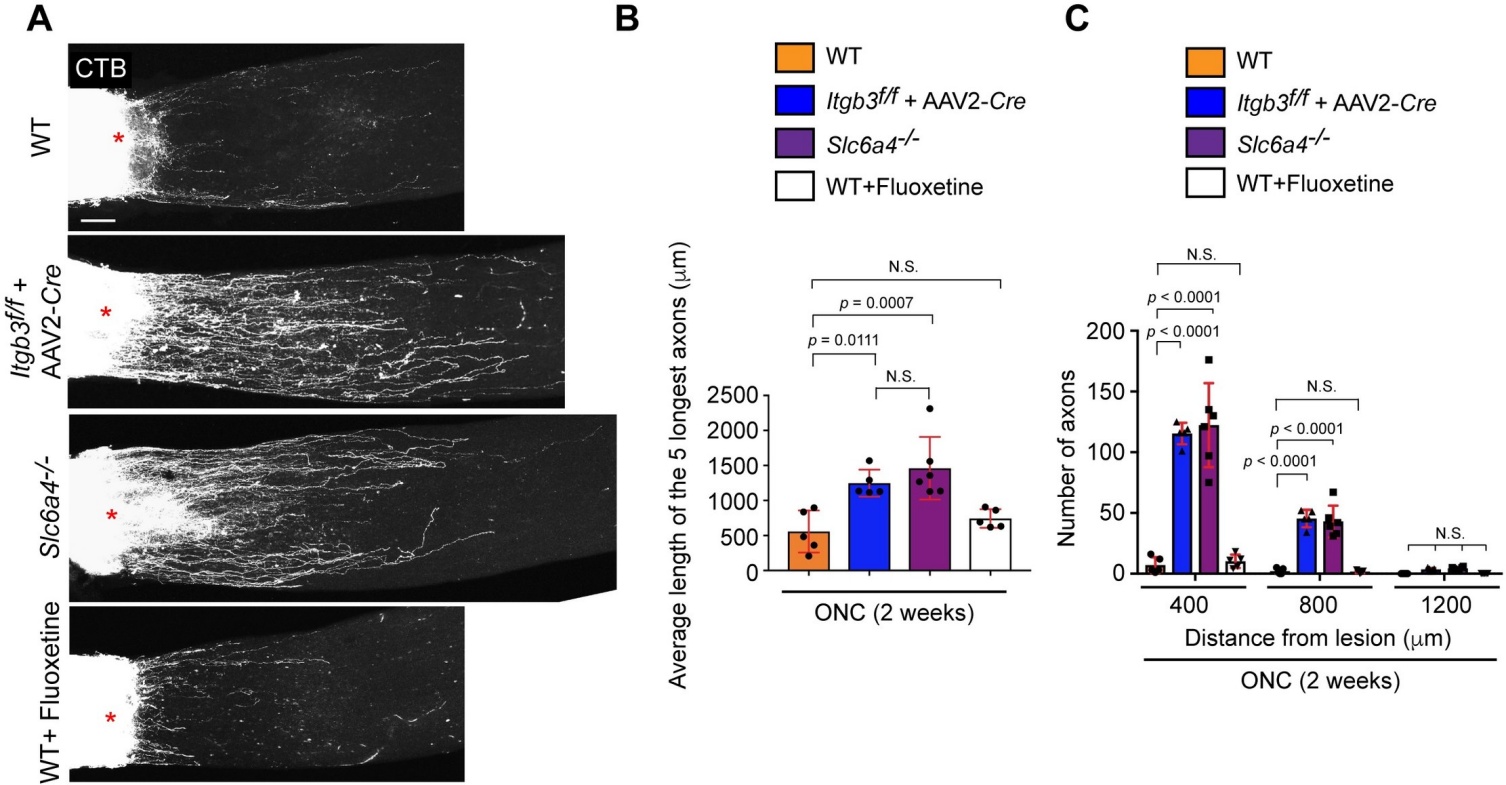
SERT and integrin β3 negatively impact retinal axon regeneration after injury. (A) CTB-labeled regenerated axons beyond the injury site (*) are observed in *Itgb3*^*flox/flox*^ mice + AAV2-GFP-*Cre* virus infected into the VT retina and *Slc6a4*^*-/-*^ mice, but not WT mice or WT mice treated with fluoxetine two weeks after ONC. (B-C) Quantitative analysis of axon regeneration in *Itgb3*^*flox/flox*^ + AAV2-GFP-*Cre*, *Slc6a4*^*-/-*^, WT mice and WT mice injected with fluoxetine (n = 5–6 mice/condition, one-way-ANOVA (*B*), two-way ANOVA (*C*)). The average of the distance of the CTB-labeled five longest axons from the lesion site *(B)* and the number of regenerated axons at 400, 800 and 1200 μm from the lesion site are shown *(C)*. Data presented as mean ± SD. N.S., not significant; Scale bar represents 100 μm.

We next investigated whether loss of SERT or integrin β3 impacts axon regeneration after ONC (Fig 5). Two weeks after ONC in WT, *Slc6a4*^*-/-*^ mutant mice and *Itgb3* ^*flox/flox*^ mice with AAV2-GFP-*Cre* virus infected into the peripheral VT retina, regenerated axons were labeled with the anterograde axonal tracer Alexa Fluor 555-conjugated cholera toxin subunit B (CTB) and fixed two days later as we previously described [35]. The whole optic nerve was cleared with a blend of two solvent-free quick tissue clearing methods that maintain the original sample size, *Clear*^*T*^ and Sca*l*eS [36,37], and CTB^+^ axons within the optic nerve were visualized by confocal microscopy. In *Itgb3* ^*flox/flox*^ retina expressing AAV2-GFP-*Cre* in the VT retina (referred to as *Itgb3*^*-/-*^) and *Slc6a4*^*-/-*^ retina two weeks after ONC, robust axon regeneration was detected when compared to WT (Fig 5A). To quantify regenerative capacity by loss of integrin β3 and SERT in the retina, we examined the average length of the five longest regenerated axons (Fig 5B) and counted axon number from the lesion site (Fig 5C). We found that the average of the five longest regenerated axons is 1247.7 μm in *Itgb3*^*-/-*^ retina and 1459.9 μm in *Slc6a4*^*-/-*^ retina while the average is only 557.2 μm in WT retina (Fig 5B). We also quantified the number of CTB^+^ axons and found that loss of integrin β3 and SERT significantly promoted axon regeneration, with axon extensions detected at up to 800 μm from the lesion (Fig 5C). Finally, we tested utilizing fluoxetine, an inhibitor of serotonin reuptake whether enhanced regeneration capacity by loss of SERT is linked with decreased serotonin activity or not. Fluoxetine was given by intraperitoneal injection one day before ONC and daily after ONC for two weeks as previously used for neuroprotection assay (Fig 3F). We found that fluoxetine had no effect on axon regeneration (Fig 5A–5C). Thus, axon regeneration ability is attenuated by SERT and integrin β3 after optic nerve injury.

### Effects of loss of SERT on gene profiles of VT RGCs after optic nerve injury

**Fig 6:**
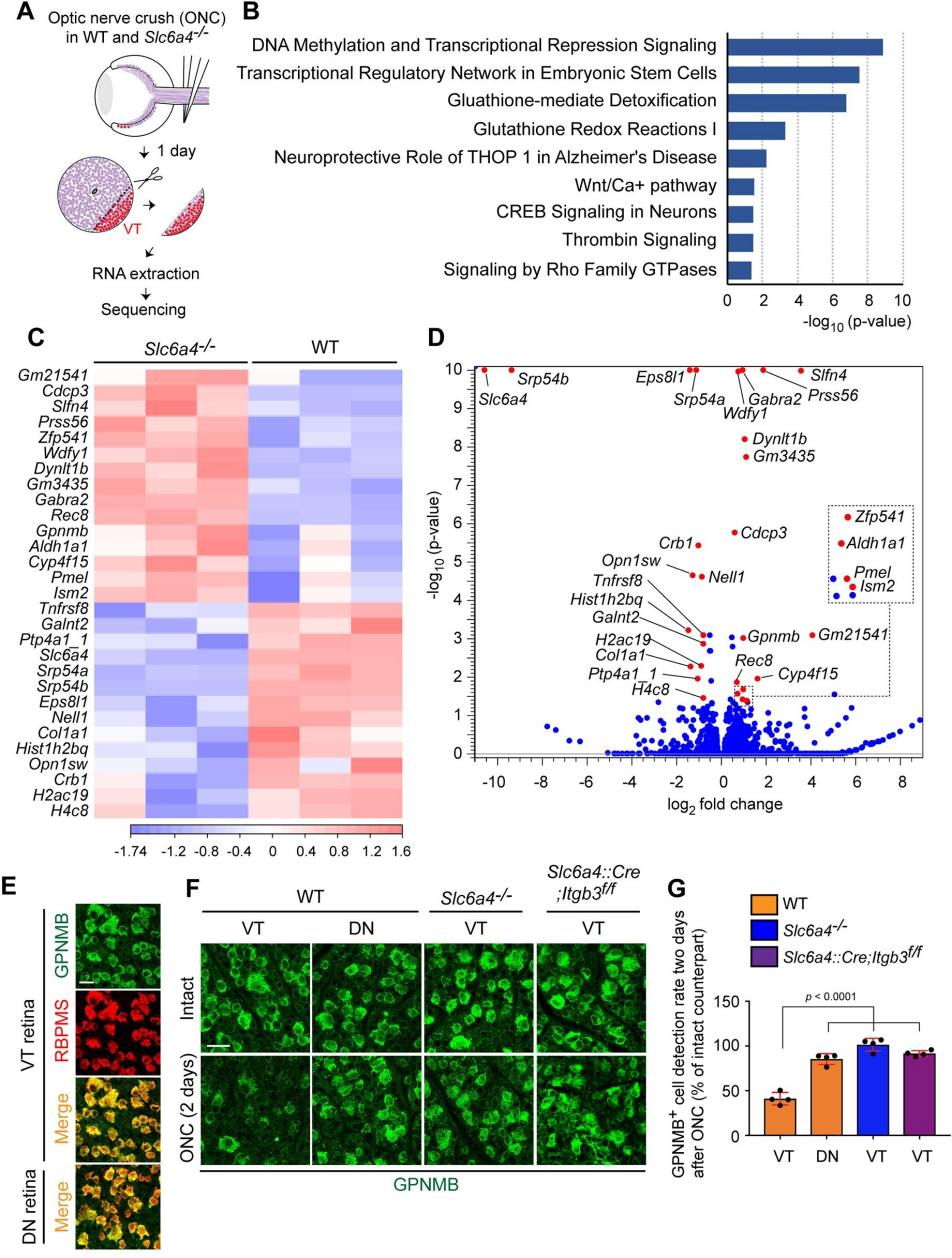
Transcriptome profiling of the VT retina in *Slc6a4*^*-/-*^ and WT mice after injury. (A) Schema of RNA preparations and sequencing for transcriptome profiling of the VT retina in *Slc6a4*^*-/-*^ and WT mice one day after ONC. (B) Enriched cellular processes and pathways in the VT *Slc6a4*^*-/-*^ retina compared to WT retina one day after ONC are shown. Bars indicate the degree of -log_10_ P value. (C-D) Heatmap *(C)* and volcano plots in red *(D)* showing the most significant 29 genes (FDR value < 0.05 and change in expression of more than 1.5-fold) in the VT retina of *Slc6a4*^*-/-*^ compared to WT mice one day after ONC (n = 3 samples/condition, each sample includes three VT retinal tissues of three *Slc6a4*^*-/-*^ or WT mice). In the heatmap *(C)*, color values indicate log2-transformed expression values shown in the color key (bottom) relative to the average expression in WT mice. The columns show individual samples from *Slc6a4*^*-/-*^ and WT mice in triplicate. (E) GPNMB is expressed in RBPMS^+^ RGCs of the VT and DN retina. (F) GPNMB^+^ cells are reduced in the VT retina of WT mice two days after ONC compared to the intact retina, while GPNMB^+^ cells in the DN retina of WT mice or the VT retina of *Slc6a4*^*-/-*^ or *Slc6a4*::*Cre;Itgb3*^*flox/flox*^ mice are still clearly detected after ONC. (G) Quantitative analysis of GPNMB^+^ cell detection rate two days after ONC compared to the intact counterpart (n = 4 mice/condition, one-way ANOVA). Data presented as mean ± SD. Scale bars represent 20 μm (E, F).

To gain new molecular insights into enhancement of VT RGC protection and axon regeneration in *Slc6a4*^*-/-*^ mice, we performed bulk RNA sequencing (RNA-seq) from the VT retina of WT and *Slc6a4*^*-/-*^ mice one day after ONC (Fig 6A). From 25,676 transcripts, 701 differentially expressed genes (DEGs) were identified that were up- or downregulated in the VT retina of *Slc6a4*^*-/-*^ mice with a threshold of p-value less than 0.05 (S1 Table). Importantly, RNA-Seq data confirmed that SERT (*Slc6a4*) expression is completely eliminated in the injured VT retina of *Slc6a4*^*-/-*^ mice (Fig 6D). The 701 DEGs include 54 genes which have been identified as genes differentially expressed in RGC subtypes after optic nerve injury (S1 Table) [16]. Moreover, the DEGs also include RGC death mediators and/or axon regeneration repressors after ONC such as *Chac1* [38], *JunD* [39], *Socs3* [40] and *Rab3c* [41] that were downregulated in the VT retina of *Slc6a4*^*-/-*^ mice compared to WT mice. In contrast, genes involved in neuroprotection and axon regeneration such as *Bmp4* [42] were upregulated in *Slc6a4*^*-/-*^ mice (S5A Fig). Moreover, ingenuity pathway analysis identified enriched cellular processes that were activated in the VT *Slc6a4*^*-/-*^ retina compared to WT retina after ONC, several of which are known to affect neuronal survival and axon regeneration (Figs 6A and S5B). For example, the glutathione-mediated detoxification pathway is involved in death/survival of RGCs and other neurons [43]. The DNA methylation signaling pathway [44], Wnt pathway [45], and Rho family GTPase pathway [46] mediate RGC axon regeneration.

We next focused on 29 DEGs with an FDR p-value less than 0.05 and with *a fold*-*change* of at least *1*.*5* (Fig 6C and 6D). Significantly affected genes, *Nell1* and *Srp54* are involved in cell death in a variety of cellular contexts [47,48]. *Wdfy1* regulates neurite outgrowth [49] and *Aldh1a1* prevents neuronal degeneration in Parkinson’s disease [50]. Among these 29 DEGs, only *Gpnmb* commonly exerts neuroprotective effects in several neurodegenerative diseases such as Amyotrophic lateral sclerosis (ALS) [51], Parkinson’s disease [52] and after brain injury [53]. Moreover, the molecular mechanisms underlying GPNMB-mediated functions in neurodegenerative diseases have been well studied. For example, in motor neurons GPNMB is neuroprotective against mutant TDP-43-indcued stress through activation of the ERK1/2 and Akt pathways [54], and these signaling pathways also contribute to retinal axon regeneration after ONC [55,56]. GPNMB also binds to CD44 expressed in astrocytes, and GPNMB reduces the ability of pro-inflammatory cytokines and induces anti-inflammatory factor, IGF-1 in astrocyte cultures [57]. Neurotoxic reactive astrocytes kill RGCs after ONC [5]. Based on previous studies demonstrating its neuroprotective effects and their mechanisms, *Gpnmb* was chosen as a top candidate, and we analyzed whether GPNMB is a regulator of RGC protection and axon regeneration after ONC. However, expression and functions of GPNMB in the retina, especially RGCs remain unclear. First, we investigated expression of GPNMB in RGCs and found that GPNMB is expressed in >95% of RGCs in the VT retina and DN retina (Fig 6E). In the VT retina of WT mice two days after ONC, this was reduced to 41.0%. In contrast, in the DN retina of WT mice or the VT retina of *Slc6a4*^*-/-*^ and *Slc6a4*::*Cre;Itgb3*^*flox/flox*^ mice, >85.4% of GPNMB^+^ RGCs were still detected even after ONC (Fig 6F and 6G). Thus, loss of SERT in the injured VT retina significantly alters molecular signatures, and one of the identified molecules, GPNMB, could be a key downstream target mediating neuroprotection and axon regeneration in the VT retina.

### GPNMB promotes RGC protection and axon regeneration after optic nerve injury

**Fig 7:**
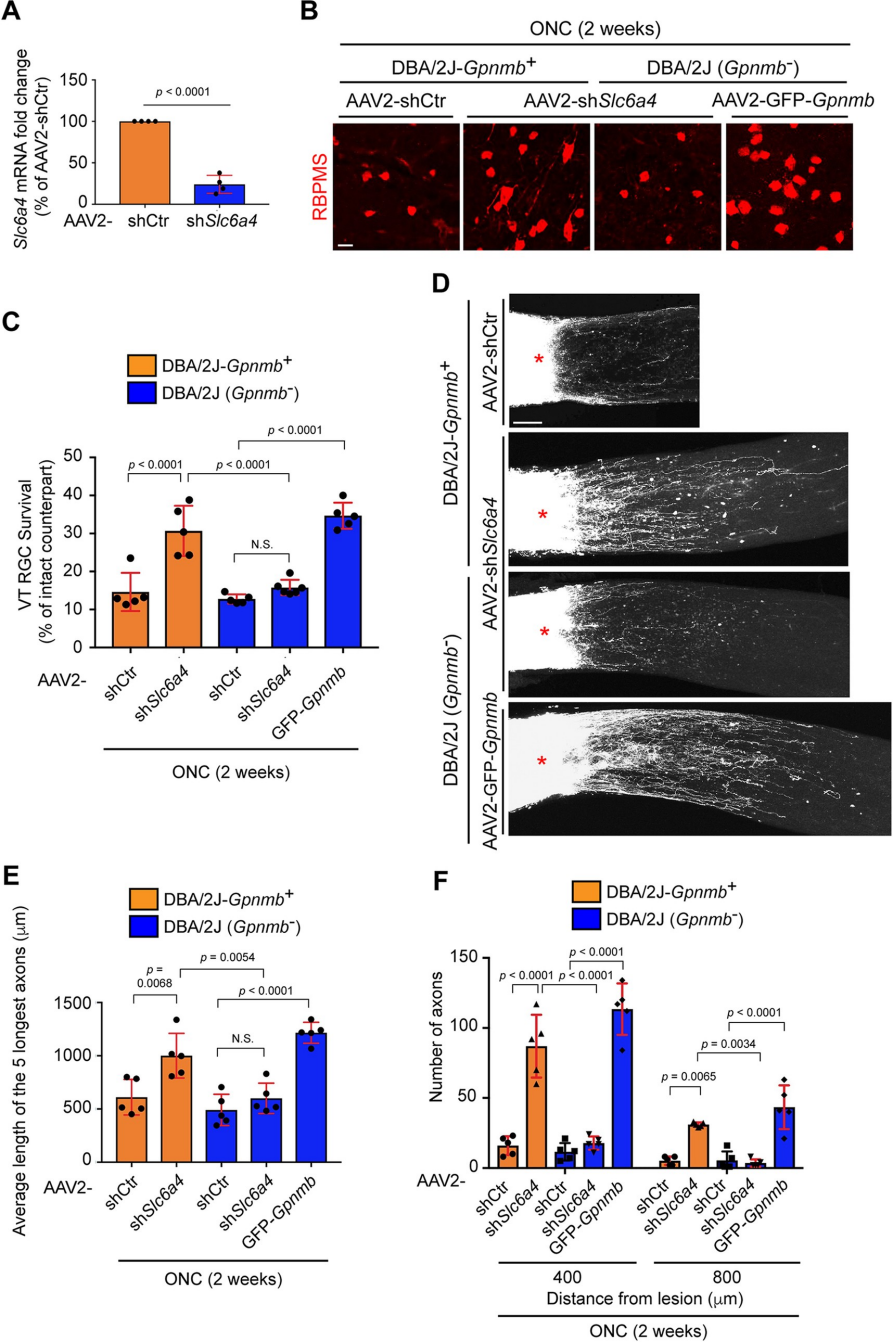
GPNMB mediates RGC survival and axon regeneration. (A) Relative *Slc6a4* mRNA expression in the VT retina two weeks after infection of AAV2-sh*Slc6a4* virus compared to AAV2-shControl (AAV2-shCtr) virus as a control (n = 4 mice/condition, two-tailed unpaired t-test). (B-C) In in DBA/2J-*Gpnmb*^+^ mice, peripheral VT RGCs are protective by AAV2-sh*Slc6a4* virus compared to AAV2-shCtr virus two weeks after ONC, while in DBA/2J mice (which carry mutations in *Gpnmb* gene), VT RGCs are not protected by AAV2-sh*Slc6a4* virus. However, Peripheral VT RGCs following AAV2-GFP-*Gpnmb* virus infection into the ventral retina of DBA/2J mice are protective after injury (n = 5 mice/condition, one-way ANOVA). (D) CTB-labeled regenerated axons beyond the injury site (*) are observed in DBA/2J-*Gpnmb*^+^ mice after AAV2-sh*Slc6a4* virus infection or DBA/2J mice after AAV2-GFP-*Gpnmb* virus infection into the ventral retina. However, DBA/2J-*Gpnmb*^+^ mice carrying AAV2-shCtr virus or DBA/2J mice carrying AAV2-sh*Slc6a4* virus show little or minimal axon regeneration two weeks after ONC, respectively. (E-F) Quantitative analysis of axon regeneration in DBA/2J-*Gpnmb*^+^ or DBA/2J mice having AAV2-sh*Slc6a4*, AAV2-shCtr or AAV2-GFP-*Gpnmb* virus in the ventral retina two weeks after ONC. The average of the distance of the CTB-labeled five longest axons from the lesion site *(E)* and the number of regenerated axons at 400 and 800 μm from the lesion site are shown *(F)*. (n = 5 mice /condition, one-way-ANOVA (*E*), two-way ANOVA (*F*)). Data presented as mean ± SD. N.S., not significant; Scale bars represent 20 μm (B) and 100 μm (D).

To directly assess whether GPNMB mediates VT RGC survival and axon regeneration via SERT, AAV2 virus-mediated shRNA silencing of *Slc6a4* (SERT) via antisense *Slc6a4* sequences under CMV promoter (referred to as AAV2-sh*Slc6a4*) and control sequences (referred to as AAV2-shCtr) were generated, and we utilized DBA/2J mouse carrying mutations in *Gpnmb* that result in a truncated and dysfunctional GPNMB protein, or the DBA/2J-*Gpnmb*^+^ mouse, carrying wild-type *Gpnmb*, as a control [58]. We examined the expression of GPNMB in RGCs of DBA/2J mice with the antibody against GPNMB and failed to detect the expression in RGCs of DBA/2J mice, but not DBA/2J-*Gpnmb*^+^ mice (S6 Fig). Although the DBA/2J mouse is broadly used as a model of inherited glaucoma, 6-10-week old DBA/2J mice for this optic nerve crush experiments do not yet display any glaucomatous phenotypes such as RGC death and intraocular pressure changes at this time [59]. Viruses were first intravitreally injected into the peripheral ventral retina to knockdown *Slc6a4* in most VT RGCs, and the knockdown efficiency assessed. We found that *Slc6a4* mRNA levels in the VT retina were reduced by 76.0% in the AAV2-sh*Slc6a4* virus-injected retina, when compared to AAV2-shCtr virus-injected retina (Fig 7A). Next, we analyzed the effects of AAV2-sh*Slc6a4* virus on VT RGC survival in DBA/2J and DBA/2J-*Gpnmb*^+^ mice two weeks after ONC (Fig 7B and 7C). In the DBA/2J-*Gpnmb*^+^ mouse, only 14.6% of VT RGCs with AAV2-shCtr virus survived after ONC, while 30.7% of VT RGCs survived when AAV2-sh*Slc6a4* virus was injected. However, AAV2-sh*Slc6a4* virus failed to attenuate VT RGC death in DBA/2J mice as AAV2-shCtr virus did (Fig 7B and 7C), suggesting that enhanced VT RGC protection and axon regeneration by loss of SERT could be meditated by GPNMB. To have the direct evidence that GPNMB regulates VT RGC protection after ONC, an AAV2 virus carrying mouse *Gpnmb* with IRES-eGFP under the CMV promoter (referred to as AAV2-GFP-*Gpnmb*) was generated, and intravitreally injected into the peripheral ventral retina (Fig 7B and 7C). AAV2-GFP-*Gpnmb* virus led to 34.7% of VT RGC survival in DBA/2J mice, while AAV2 control virus showed only 12.8% of VT RGC survival. Thus, GPNMB acts as a regulator of neuroprotection in the VT retina after ONC.

Next, we examined whether SERT and GPNMB mediate retinal axon regeneration after ONC in DBA/2J and DBA/2J-*Gpnmb*^+^ mice (Fig 7D). We examined the average of the five longest regenerated axons (Fig 7E) and counted axon number from the lesion site (Fig 7F) two weeks after ONC. We found that AAV2-sh*Slc6a4* virus resulted in robust axon regeneration in DBA/2J-*Gpnmb*^+^ mice, but not DBA/2J mice. However, AAV2-GFP-*Gpnmb* virus significantly induced axon regeneration in DBA/2J mice (Fig 7D–7F). We also examined the effects of GPNMB on dorsal RGC survival and axon regeneration two weeks after ONC following AAV2-GFP-Gpnmb virus infection into the peripheral dorsal retina of DBA2/J mice (S7 Fig). GPNMB also promoted dorsal (DT and DN) RGC survival (S7B and S7C Fig) and axon regeneration (S7D–S7F Fig) compared to control virus. Thus, RGC survival and axon regeneration are promoted by maintaining GPNMB expression after ONC, and SERT-deficient VT RGCs retain GPNMB expression and its functions even after ONC.

## Discussion

Selective neuronal vulnerability is a common pathological feature in many neurodegenerative diseases and after optic nerve injury. Here, we focused on identifying acute neurodegenerative mechanisms based on the early susceptibility of peripheral ventrotemporal (VT) RGCs to degeneration after ONC. We found that neurodegenerative and regeneration-inhibitory signals conveyed from the optic nerve to VT RGCs after ONC are mediated by SERT-integrin β3 molecular axis, which acutely reduces GPNMB expression. Taken together, our study identifies a novel molecular mechanism mediating the region-specific neuronal vulnerability of RGCs to acute injury.

Peripheral VT RGCs possess anatomically and molecularly distinct features compared to RGCs in other regions of the mouse retina [21]. In mouse, the peripheral VT retina includes RGCs projecting ipsilaterally, which comprise <5% of all RGCs, while the remainder of the RGCs project contralaterally. In humans, the temporal retina possesses ipsilateral RGCs which constitute 45% of all RGCs. Our results revealed that the peripheral VT RGCs, especially ipsilaterally-projecting RGCs, are more vulnerable to degeneration than RGCs in other regions. It has been reported that SERT is expressed in ipsilateral RGC axons during development and at postnatal stages [26–28]. We found that SERT expression is barely detected in intact adult VT axons, but elevated SERT expression on VT axons is observed after ONC. In vivo analysis using *Slc6a4*^*-/-*^ mice has shown that SERT mediates RGC vulnerability and axon regeneration in the VT retina. Several studies have shown that local translation of specific molecules on axons after nerve injury and in neurodegenerative diseases leads to regulation of neuronal survival/death and axon regeneration [60,61]. Thus, SERT expression on axons after optic nerve injury could be regulated by the local translation machinery.

Intriguingly, integrin β3, a molecule mediating SERT-induced functions in other systems [33,34], is also upregulated in RGCs and on retinal axons after ONC. RNA-seq analyses in zebrafish RGCs show elevated expression of *itgb3* (especially, *itgb3b*) in the injured RGCs after optic nerve injury when compared to intact RGCs [62]. However, RNA-seq analyses in the mouse retina or RGCs failed to show the elevated expression of *itgb3* after injury [16,63], while we found that integrin β3 protein expression is elevated in injured RGCs and axons (Fig 4). It could be possible that surface expression of integrin β3 protein may be elevated in RGCs and their axons in response to injury-induced molecules and ligands since surface expression of integrin family members is altered in the presence of their ligands [64–66]. Since these observations are still unclear, further studies are needed to determine the mechanisms. We also found that loss of integrin β3 led to VT RGC survival and axon regeneration as observed in *Slc6a4*^*-/-*^ mice. It is intriguing that integrin β3 effects seem to be specific to VT RGCs. It is known that activated integrin β3 enhances SERT-dependent platelet functions [33]. Thus, integrin β3 may serve as a mediator in enhancing SERT-induced functions, but not as a determinant in executing RGC death after optic nerve injury.

SERT normally controls the concentration of extracellular free 5-HT at the presynaptic terminal to regulate SERT-dependent activity, which then modulates serotonergic neurotransmission and affects not only visual and but also other neuronal circuit formation during development and postnatal stages [27,29,32,67,68]. Several reports have shown that serotonin positively and negatively regulates axon regeneration and mediates neuronal survival depending on expression and location of its receptors, downstream signaling pathways and/or animal/injury models [69–73]. The evidence implies that enhanced retinal axon regeneration and neuroprotection in the *Slc6a4*^*-/-*^ VT retina after ONC could be triggered by (i) reduced serotonin reuptake and/or (ii) binding of excess extracellular free 5HT to serotonin receptors expressed in VT RGCs. The dose (10mg/kg, IP, daily) of fluoxetine was utilized in this study since it has been reported to show robust effects on stress-related behavior [74], several motor behaviors [75] and retinal axon formation from RGCs to the superior colliculus [28,76]. However, we found that fluoxetine, a serotonin reuptake inhibitor had no neuroprotective and regenerative effects in the VT retina after ONC while *Slc6a4*^*-/-*^ mice showed these effects (Figs 3 and 5). Intriguingly, we also found that daily treatments of fluoxetine one day before and for five days after ONC reduced the number of microglia/macrophages at the lesion site compared to control, saline, while *Slc6a4*^*-/-*^ and WT mice displayed a similar number of microglia/macrophages at the lesion site (S3 Fig). In fact, such inhibitory effects of fluoxetine have been previously reported in microglial cultures [77] and after ischemia [78] and brain injury [79] in vivo. Thus, whether SERT mediates RGC neuroprotection and axon regeneration through serotonin-dependent or independent mechanisms is not conclusive yet since fluoxetine affects the whole retinal environment.

How does SERT mediate degenerative and regeneration-inhibitory signals? A precedent has shown that SERT binds to platelet-derived growth factor receptor (PDGFR), mediates phosphorylation of PDGFR and serves as a modulator that facilitates transmission of signals from PDGFR to its associated signaling molecules upon the presence of its ligand, PDGF [80]. This functional model could also be applied to the mechanisms of SERT-integrin β3-induced functions since (i) loss of SERT led to decreased numbers of phosphorylated integrin β3^+^ VT RGCs, (ii) loss of SERT and integrin β3 led to VT RGC protection and axon regeneration, (iii) a downstream molecule, GPNMB is normally downregulated in WT VT RGCs after ONC, but not in *Itgb3*^*-/-*^ or *Slc6a4*^*-/-*^ RGCs, and (iv) GPNMB promotes neuroprotection and axon regeneration in the VT retina as observed in *Itgb3*^*-/-*^ or *Slc6a4*^*-/-*^ VT RGCs. Taken together, SERT and integrin β3 could play a critical role in mediating degenerative and regenerative-inhibitory signal transduction after ONC. Further studies are needed to identify ligands mediating downstream signals via integrin β3-SERT after ONC, leading to development of new therapeutic strategies after ocular trauma.

It remains an open question as to how the SERT-integrin β3-GPNMB molecular axis mediates axon regeneration and neuroprotection. Our RNA-seq data could provide clues to this process. For instance, *Dynlt1b* (also known as Tctex-1), which was upregulated in the *Slc6a4*^*-/-*^ VT retina, has been reported to enhance axon outgrowth by modulating Rho signaling pathways and actin/microtubule dynamics [81,82]. *Nell1* and *Srp54*, which were downregulated in the *Slc6a4*^*-/-*^ VT retina, are involved in cell death in a variety of cellular contexts [47,48]. Moreover, other potential downstream candidates could be factors functioning as RGC death mediators and/or axon regeneration repressors after ONC such as *Chac1* [38], *JunD* [39], *Socs3* [40] and *Rab3c* [41] that were downregulated in the VT retina of *Slc6a4*^*-/-*^ mice compared to WT mice (S1 Table). Thus, some or all of these molecules could regulate axon regeneration and/or neuroprotection after injury as downstream components of the SERT-mediated molecular axis.

Our RNA-seq data show that *Pmel* is upregulated in *Slc6a4*^*-/-*^ VT retina compared to WT VT retina after optic nerve injury. PMEL (encoded by *Pmel*) is normally expressed in the pigment cells such as the RPE, and the melanosomes in *Pmel*^−/−^ RPE showed abnormal melanin aggregates and poorly preserved membranes, suggesting PMEL maintains normal pigment production in the RPE [83]. Intriguingly, melanin synthesis in the RPE coincides with neurogenesis of RGCs during development, and disruption of pigmentation in the RPE leads to delayed neurogenesis of ipsilateral RGCs in the VT retina [84]. Moreover, the pigmented and albino RPE differentially express signaling molecules such as Wnt2b, which regulates ipsilateral RGC production [85]. Thus, elevated expression of PMEL in the RPE of *Slc6a4*^*-/-*^ VT retina after optic nerve injury could facilitate pigment production and maintain RPE integrity, resulting in altered expression of signaling molecules that may affect neuroprotection and axon regeneration via specific molecular interactions between the RPE and VT RGCs.

The magnitude of RGC vulnerability after optic nerve injury and in ocular diseases varies depending on the RGC subtypes [10–14,16]. A resistant RGC subtype, αRGC is expressed evenly in all retinal regions (S1 Fig). However, intrinsically photosensitive retinal ganglion cells (ipRGCs) are a rare subpopulation of RGCs (<5% of all RGCs, 1600–1700 cells in the entire retina) and resistant to optic nerve injury [13]. ipRGCs (M1 and M2 cells) are sparsely located in the entire retina, but the larger number of ipRGCs is located in the dorsal retina compared to the ventral retina, while there are no differences of the number between the temporal and nasal retina [86]. Thus, more enriched distribution of vulnerable or resistant RGC subtypes such as ipRGCs in the specific regions could also contribute to the differential RGC vulnerability after injury.

Our RNA-seq data also have shown that expression levels of several RGC subtype-specific markers are altered in the VT retina of *Slc6a4*^*-/-*^ mice after ONC compared to WT retina such as *Col25a1* (1.16-fold increase) a marker of D- and V-ooDSGCs [87] and *Ebf3* (1.17-fold decrease), a marker of F-RGCs [16]. Moreover, genes expressed in susceptible RGC subtypes [16] such as *Tac1* (1.38-fold increase), *Crhbp* (1.96-fold increase) and *Gabra2* (1.95-fold increase) are also affected in the VT retina of *Slc6a4*^*-/-*^ mice after ONC (S1 Table). Thus, these additional data show that loss of SERT in the VT retina protects RGCs from injury.

Glaucoma is a devastating neurodegenerative disease that affects ~64 million people. Intriguingly, DBA/2J mice, one of the major animal models used to study glaucoma, retain genetic mutations in the *Gpnmb* gene, while DBA/2J-*Gpnmb*^+^ mice display no glaucomatous phenotypes such as IOP elevation and RGC death [88]. Loss of *Gpnmb* leads to iris pigment dispersion, thereby resulting in elevated IOP [58,88,89]. Our results demonstrate that expression of GPNMB in RGCs is also neuroprotective after ONC. Thus, maintaining GPNMB expression in glaucomatous RGCs could halt RGC death and optic nerve degeneration, and GPNMB could be a common therapeutic target enhancing neuroprotection and axon regeneration in neurodegenerative diseases and after nerve injury.

## Materials and methods

### Ethics statement

All animal experiments were performed according to the regulatory guidelines of the University of Pittsburgh School of Medicine Institutional Animal Care and Use Committee (IACUC; protocol number 18032315, 21039031).

### Animals

Animals used are described as follows: C57BL/6J mice (referred to as WT, Jackson Laboratory, 000664, RRID:IMSR_JAX:000664), *Slc6a4*^*-/-*^ mice (Jackson Laboratory, 008355, RRID:IMSR_JAX:008355), *Itgb3*^*flox/flox*^ mice (Jackson Laboratory, 028232, RRID:IMSR_JAX:028232), B6.FVB(Cg)-Tg(Slc6a4-cre)ET33Gsat/Mmucd (referred to as *Slc6a4*::*Cre*, RRID:MMRRC 031028-UCD to which they were donated by Dr. Nathaniel Heintz), B6.Cg-Gt(ROSA)26Sor^tm6(CAG-ZsGreen1)Hze^/J mice (referred to as *R26R*^*ZsGreen*^, Jackson Laboratory, 007906, RRID:IMSR_JAX:007906), DBA/2J mice (Jackson Laboratory, 000671, RRID:IMSR_JAX:000671) and DBA/2J-*Gpnmb*^+^/sjJ mice (referred to as DBA/2J-*Gpnmb*^+^, Jackson Laboratory, 007048, RRID:IMSR_JAX:007048) were purchased and used for experiments. In particular, *Slc6a4*::*Cre;Itgb3*^*flox/flox*^ mice and *Slc6a4*::*Cre;Itgb3*^*+/+*^ mice as control were generated by intercrossing *Slc6a4*::*Cre;Itgb3*^*flox/+*^ mice. *Slc6a4*::*Cre;R26R*^*ZsGreen*^ mice were generated by crossing *Slc6a4*::*Cre* mice with *R26R*^*ZsGreen*^ mice. All mice were housed in a pathogen-free barrier facility. Mice were maintained in a 12 h light/dark cycle with access to standard laboratory chow and water *ad libitum*. Both sexes were used for all experiments.

### Optic nerve crush

8-10-week-old animals were anesthetized with ketamine/xylazine. To expose the optic nerve, the dural sheath surrounding the optic nerve in the left eye was carefully incised. The optic nerve in the left eye was then crushed using #5 Dumont forceps, applying pressure for 5s ~1 mm behind the optic disc. The right eye was intact (uninjured) retina. Following various time points after optic nerve crush, animals were humanely euthanized, perfused with 4% paraformaldehyde (PFA) following PBS, and the head was cut and post-fixed in 4% PFA overnight at 4°C and washed with PBS for further analysis. For analysis of effects of fluoxetine, fluoxetine hydrochloride was purchased from Sigma-Aldrich (#1279804), prepared fresh daily and given by intraperitoneal injection (10mg/kg) one day before ONC and daily after ONC for two weeks as previously utilized for analysis of retinal axon projection [28,76]. Time points for RGC survival/death and axon regeneration analysis are indicated in each figure legend and described in the main text.

### Intravitreal injection

For intravitreal injection of all materials described below, glass micropipettes made by a micropipette puller was used. For axon regeneration assay, 2 μl of 2 μg/μl of Alexa Fluor 555 conjugated cholera toxin β subunit (CTB) (ThermoFisher, C22843) were injected into the central retina 2 days before euthanasia. 1 μl of AAV2-CMV-GFP-*Cre* virus (1x10^13 GC/ml, VECTOR BIOLABS, 7016), AAV2-CMV-GFP-sh*Slc6a4* virus (target sequences: GCCCTCTGTTTCTCCTGTTCA) [90] (1x10^13 GC/ml, VIROVEK, custom-made), AAV2-CMV-GFP-shControl virus (control sequences: GTCTGTTTCCCTCGCTACTCT) (1x10^13 GC/ml, VIROVEK, custom-made), AAV2-CMV-mouse *Gpnmb*-IRES-eGFP (2.2x10^12 GC/ml, VECTOR BIOLABS, 260538) was slowly intravitreally injected into the peripheral ventral or dorsal retina of 6-week-old WT or transgenic mice.

### Immunohistochemistry

For whole mount immunofluorescent staining of the injured and intact retina, after removal of the cornea, lens and retinal pigment epithelium, the ventral retina was cut with microscissors, creating a small incision as a marker for orienting the retina. The injured and intact retina were dissected out from the left and right retina, respectively and incubated in different tubes for 1 hour at 4°C in PBS containing 0.4% Triton X-100 (PBST). Each retina was incubated with primary antibodies in PBST overnight at 4°C and washed with PBST three times. The retina was incubated with secondary antibodies in PBST overnight at 4°C and washed with PBST three times. The dorsal, nasal and temporal regions in the intact and injured retina were also cut with micro scalpels on the microscope slide and mounted with Fluoro-Gel mounting medium (Electron Microscopy Sciences, 17985–11). For cryosectioning, the retina or optic nerve was incubated in 30% sucrose PBS for 48 h at 4°C, and then embedded with Tissue-Tek O.C.T. and 14 μm retinal or optic nerve cryosections were prepared and immunostained described above. For immunostaining with integrin β3 and SERT (Millipore, AB9726) antibodies, PBS was used instead of PBST during the immunostaining process and sections were incubated with the primary antibodies overnight at room temperature and with the secondary antibodies for 4 hours at room temperature. We also tested goat SERT (1:500, Abcam, ab130130) antibody in *Slc6a4*::*Cre;R26R*^*ZsGreen*^ and WT mice, and the immunoreactivity was somehow diminished in WT, but not *Slc6a4*::*Cre;R26R*^*ZsGreen*^ mice so that we decided that the antibody was not appropriate in this study. For immunostaining with GPNMB antibody in the whole mount retina, PBS containing 0.04% Triton X-100 was used. For immunostaining with phosphorylated-integrin β3 Antibody (pY759.7A) in the whole mount retina, the retina was incubated in cold methanol for 10 mins, and PBS was used for washing and during further immunostaining processes. Immunolabeling was performed with the following primary antibodies: mouse βIII-tubulin (2G10, 1:500, Lifetechnology, MA1118, RRID:AB_309804), guinea pig RBPMS (1:500, Millipore, ABN1376, RRID:AB_2687403), chick GFP (1:600, Abcam, ab13970, RRID:AB_300798), goat osteopontin (OPN) (1:200, R&D Systems, AF-808, RRID:AB_2194992), rabbit SERT (1:1000, Millipore, AB9726, RRID:AB_612176), mouse phosphorylated-integrin β3 (pY759.7A) (1:200, SantaCruz, sc-136458, RRID:AB_10650118), mouse integrin β3 (1:200, ThermoFisher, 16-0611-82, RRID:AB_468984), rabbit neurofilament L (1:500, Millipore, AB9568, RRID:AB_11213875), goat GPNMB (1:300, R&D system, AF2330, RRID:AB_2112934) and mouse Iba1(1:200, Millipore, MABN92, RRID:AB_10917271). Secondary antibodies were used at 1:500 and purchased from Lifetechnology: donkey anti-Rabbit IgG 647 (A-31573, RRID:AB_2536183), goat anti-Mouse IgG 488 (A-11001, RRID:AB_2534069), goat anti-Rabbit IgG 594 (A-11037, RRID:AB_2534095), donkey anti-Mouse IgG 647 (A-31571, RRID:AB_162542), goat anti-Guinea Pig IgG 647 (A21450, RRID:AB_141882), goat anti-Guinea Pig IgG 488 (A-11073, RRID:AB_2534117), donkey anti-Goat IgG 488 (A-11055, RRID:AB_2534102), goat anti-Chick IgG 488 (A11039, RRID:AB_142924), donkey anti-Mouse IgG 594 (A21203, RRID:AB_141633). DAPI was used at 1:500 and purchased from ThermoFisher (D1306, RRID:AB_2629482). ZsGreen^+^ VT RGCs and axons were detected with intrinsic fluorescence of ZsGreen protein without the antibody.

### Imaging

Whole optic nerve labeled with CTB, or whole retina or cryosections after immunostaining were imaged on Olympus Fluoview 1000 laser scanning microscope with Fluoview software, a 20× objective lens (PlanSApo, NA = 0.85), a 40× objective lens (UPlanFL N NA = 1.3) or a 60x objective lens (PlanApo NA = 1.42).

### Clearing optic nerve

Tissue clearing reagents based on the combinations of previously described clearing methods, *Clear*^*T*^ [37] and Sca*l*eS [36] were prepared. *Clear*^*T*^/Sca*l*eS clearing solutions are composed of formamide (Fisher scientific, F84-1), methyl-β-cyclodextrin (Fisher scientific, 50-144-1446), γ-cyclodextrin (Fisher scientific, AC229905000), D-(-) Sorbitol (Fisher scientific, 50-489-009), and Triton X-100 (Sigma-Aldrich, X100). Stock solution I (10 mM methyl-β-cyclodextrin and 10 mM γ-cyclodextrin in ddH_2_O) and stock solution II (20% D-(-) Sorbitol and 0.2% Triton X-100 in ddH_2_O) were prepared and stored at room temperature. Fixed CTB 555-labeled optic nerve was incubated in the 1.5ml tube containing 50 μl of the stock solution I, 250 μl of the stock solution II and 200 μl of PBS at room temperature overnight. The optic nerve was then incubated in the tube containing 200 μl of formamide and 300 μl of PBS at room temperature for two hours, and in the tube containing 200 μl of formamide and 300 μl of ddH_2_O at room temperature for two hours. Finally, the optic nerve was incubated in 500 μl of formamide for 30 mins—one hour at 42°C, and mounted on the microscope slide with the cover glass and ~100 μl of formamide. 1 hour after mounting, CTB-labeled regenerated retinal axons were imaged on the confocal microscope with a 20x objective lens and the whole optic nerve imaging was taken in ~80 images, 2 μm steps. Each image was stitched and the whole optic nerve image was acquired.

### Quantification

RGC survival analysis in the whole mount retina was performed by counting the number of βIII-tubulin^+^ or RBPMS^+^ RGCs in a 400 μm x 600 μm area in four quadrants, ventrotemporal (VT), ventronasal (VN), dorsotemporal (DT) and dorsonasal (DN) of the peripheral retina, the closest region to the peripheral edge as described in Fig 1A or in the middle VT retina, the region in between the peripheral edge of the VT retina and the optic disc as described in Fig 1D. After counting the number of RGCs in each corresponding region of the injured retina from the left eye and intact contralateral retina from the right eye in the same animal, RGC survival rate in each region (%) each animal was calculated by dividing the RGC number in the injured retina by the number in the intact retina.

Osteopontin^+^ (OPN^+^) RGCs (%) shown in S1 Fig in the whole mount retina was calculated by dividing the OPN^+^/βIII-tubulin^+^ number by the βIII-tubulin^+^ number in a 400 μm x 600 μm area of each quadrant of the intact naïve peripheral retina each animal.

For quantification of *Slc6a4-Cre*-derived ZsGreen expression in intraretinal axons in the VT retina, the pixel intensity of ZsGreen^+^ axons in a 400 μm x 400 μm area in the VT retina adjacent to the optic disc as shown in Fig 2G, was measured with ImageJ software. For analysis of endogenous SERT protein expression on VT axons, 30 μm cryosections were prepared. The pixel intensity of SERT in 200 μm long optic nerve bundles adjacent to the optic disc as shown in Fig 2I, was measured with ImageJ software. Fold change of SERT expression in retinal axons after injury was obtained by dividing the pixel intensity in the injured VT axons by that in the intact VT axons from the same animal.

For integrin β3 expression in the retinal and/or optic nerve cryosections, 14 μm retinal or optic nerve cryosections were prepared and immunostained with anti-integrin β3 antibody and the secondary antibody and images of injured and intact retina and/or optic nerve were taken with the same setting of the inverted confocal microscope. For quantification of integrin β3 expression in the injured or intact optic nerve cryosections, the pixel intensity of integrin β3^+^ optic nerve in a 200 μm-optic nerve column adjacent to the lesion site in injured optic nerve or at the same location in the intact optic nerve was measured with ImageJ software. Fold change of integrin β3 expression in the optic nerve after injury was obtained by dividing the pixel intensity in the injured optic nerve by that in the intact nerve from the same animal. The analysis was performed on two non-consecutive cryosections per animal and the average of fold change was obtained per animal. For counting integrin β3^+^ RGCs in the VT retina, RBPMS^+^ RGCs showing bright integrin β3^+^ fluorescent signal intensity were counted after evaluating the threshold level compared to the background. The analysis of integrin β3^+^ RGCs was performed on two non-consecutive cryosections per animal and the average of the number of those RGCs was obtained per animal. Phosphorylated-integrin β3^+^ cells in the RGC layer of the whole mount retina were counted in a 200 μm x 200 μm area in the VT intact or injured retina of WT and *Slc6a4*^*-/-*^ mice after evaluating the threshold level compared to the background, and the total number of phosphorylated-integrin β3^+^ cells was obtained per animal.

GPNMB^+^ cells in the whole mount retina were counted in a 300 μm x 300 μm area in the VT or DN intact or injured retina and GPNMB^+^ cell detection rate two days after ONC (%) was calculated by dividing the GPNMB^+^ cell number in the injured retina by the number in the intact retina.

Area where activated Iba1^+^ microglia/macrophages accumulated in the optic nerve five days after ONC was measured by Fiji and normalized to WT mice treated with PBS.

For quantification of regenerative capacity in mutants and WT mice, merged stack of ~80 images of CTB-labeled regenerated axons in the optic nerve was prepared with Fiji software. For quantification of the average length of the five longest regenerated axons, the distance of the five longest axons from the lesion site was measured with ImageJ and the average of the longest axons was obtained each animal. In case of some long-regenerated axons turning back to the retinal side, the distance between the lesion site and the turning site was measured. For counting axon number from the lesion site, a line transecting the merged stack image at 400, 800 or 1200 μm from the lesion site was drawn, and the number of the CTB-labeled axons crossing the transection line was counted per animal.

### TUNEL

RGC death two and three days after ONC was confirmed with the TUNEL assay on 14 μm cryosections from snap-frozen eyeballs. Fluorescein *In situ* Cell Death Detection kit (Millipore, S7110) was utilized according to the manufacture’s protocol. Four sections including VT and DT regions per mouse were used, and TUNEL^+^ cells in the RGC layer within 300 μm from the peripheral edge of VT and DT retina were counted. The average of TUNEL^+^ cells in the RGC layer per section was assessed and evaluated depending on locations and time postinjury.

### Quantitative RT-PCR

The whole injured and intact retina were dissected out in cold RNase-free PBS. The peripheral VT region of the injured and intact retina in Fig 7A was cut with micro scalpels, and collected into the 1.5 ml tube. For total RNA preparation, cells were lysed, and RNA was isolated using TRIzol (ThermoFisher, 12183555), chloroform (Fisher scientific, C298-500) and 2-propanol (Fisher scientific, A416-500). RNA (200 ng) was reverse-transcribed to cDNA. cDNA synthesis was performed using the High-Capacity RNA-to-cDNA Kit (ThermoFisher, 4387406). Quantitative PCR was performed in duplicate using iTaq Universal SYBR Green (BioRad, 1725121) and the CFX384 Touch Real-Time PCR Detection System (BioRad). Results are presented as linearized Ct values normalized to *Gapdh* gene. The results of qRT-PCR in the injured retina were normalized to the mean value of the intact retina for each experiment, and experiments were repeated three times with specific primers described in PrimerBank: *Slc6a4* (ID: 7110639a1, Forward: 5’-TATCCAATGGGTACTCCGCAG-3’, Reverse: 5’-CCGTTCCCCTTGGTGAATCT-3’) and *Gapdh* (ID: 6679937a1, Forward 5’-AGGTCGGTGTGAACGGATTTG-3’, Reverse: 5’- TGTAGACCATGTAGTTGAGGTCA- 3’).

### RNA sequencing

One day after optic nerve crush in three WT mice and three *Slc6a4*^*-/-*^ mutants, three peripheral VT retinae from WT mice or *Slc6a4*^*-/-*^ mutants were pooled in the same tube after dissection, and total RNA was extracted. This sample preparation was conducted in triplicates for both WT and *Slc6a4*^*-/-*^ mutants. RNA was isolated using TRIzol RNA isolation reagent (ThermoFisher) and RNA quality was verified by the University of Pittsburgh Health Genomics Core using Agilent Tapestation RNA and Qubit Fluorometric Quantification. RNA samples with the RNA integrity number of (RIN) >7 were utilized. Briefly, cDNA was synthesized with SuperScript IV and strands underwent Adenylation of 3’ ends followed by adapter ligation and library amplification with indexing. Library preparation was conducted with TruSeq Total RNA library kit (Illumina) using at least 1 μg of total RNA following the manufacture’s instruction. Modifications during the library preparation include rRNA reduced RNA cleaned up was done with AMPureXP beads and library amplified with 13 cycles of PCR and cleaned up with 37μl AMPureXP beads. Libraries were sequenced using NextSeq500 (Illumina) with 75 base paired end reads resulting in 40M reads per sample. FASTQ files were analyzed using CLC Genomics Grid Workbench v20.0. For RNA sequencing analysis, sequenced reads were aligned to the mouse genome (GRCm38). Read counts were generated and differentially expressed genes were obtained using CLC Genomic Workbench v20.0. Transcripts with at least 10 read events in two samples of either WT or *Slc6a4*^*-/-*^ mutants and with a p-value lower than 0.05 were collected so that 701 transcripts out of 25,676 transcripts were utilized for further analysis. For further expression shown in heatmaps and volcano plots and functional analysis, 29 transcripts which show the false discovery rate (FDR) p-value (<0.05) and a fold change higher than 1.5 were selected. Heatmaps and volcano plots were generated using CLC Genomic Workbench v20.0.

### Statistics and reproducibility

Sample sizes were based on past experience with studies in mouse models of retinal axon crush and other CNS diseases where phenotypes are obvious, and this range of the number per experiment can be also found in our previous published paper [35] and other literature [3–9]. All data were analyzed, and graphs were constructed using Image J, Fiji and Microsoft Excel. All error bars represent the standard deviation (SD), and statistical analysis was determined using unpaired two-tailed Student’s t-test, one-way ANOVA or two-way ANOVA followed by the Tukey’s post hoc test, as indicated in the figure legends associated with each figure. A p-value < 0.05 was considered significant. All statistical tests were performed using GraphPad Prism 7. The number of samples or animals used in each experiment was also indicated in each plot in the figure. To ensure the appropriate and fair data analysis, especially RGC counting, T.K. took all images of RGCs in different retinal regions in all experimental mice, and T.K. sent images with only codes (#1, #2, #3.) to R.K., D.A. or S.M. randomly and they checked and counted cells using Fiji, and they provided the total number in each image. T.K. collected all data and performed statistical analysis in each experiment. T.K. then notified engaged scientists of the image information such as regions and mouse lines where they blindly counted cells.

## Supporting information

S1 Fig Distribution of αRGCs in the intact peripheral retina. (A) Distribution of osteopontin (OPN)^+^ RGCs in four quadrants of the peripheral intact retina. Osteopontin (OPN)^+^/βIII-tubulin^+^ RGCs are detected in both VT and DN retina. (B) Quantitative analysis of osteopontin (OPN)^+^ RGCs (%) in four quadrants of the intact peripheral retina (n = 4, one-way ANOVA). Data presented as mean ± SD. N.S., not significant; Scale bar represents 20 μm.
(TIF)

S2 Fig Analysis of the number of RGCs in *Slc6a4*^*-/-*^ mice compared to WT mice. Quantification of βIII-tubulin^+^ RGCs in four quadrants of the intact peripheral retina in *Slc6a4*^*-/-*^ and WT mice (n = 5/condition, two-way ANOVA). Data presented as mean ± SD; N.S., not significant.
(TIF)

S3 Fig Fluoxetine shows a reduced number of microglia/macrophages at the lesion site five days after injury. (A-B) Five days after ONC, WT mice treated with fluoxetine show a reduced area of Iba1^+^ activated microglia/macrophages at the lesion site in the optic nerve compared to WT or *Slc6a4*^*-/-*^ mice with saline (n = 4/condition, one-way ANOVA). Data presented as mean ± SD. Scale bar represents 100 μm.
(TIF)

S4 Fig Integrin β3 is not involved in dorsal RGC death after injury. (A) One day after ONC, integrin β3 is upregulated in DN RGCs (arrows). (B) Schema of deletion of *Itgb3* in the dorsal retina of *Itgb3*^*flox/flox*^ mice after intravitreal injection of AAV2-GFP-Cre virus and RBPMS^+^ RGC survival analysis in the peripheral DN WT or *Itgb3* ^*flox/flox*^ + AAV2-GFP-Cre retina two weeks after ONC. (C-D) DN RGCs in WT and *Itgb3* ^*flox/flox*^ + AAV2-GFP-Cre retina are similarly vulnerable to injury (n = 4/condition, two-tailed unpaired t-test). Data presented as mean ± SD. N.S., not significant; Scale bars represent 20 μm.
(JPG)

S5 Fig Altered pathways and genes in the VT retina of *Slc6a4*^*-/-*^ mice compared to WT mice after optic nerve injury. (A) In the VT retina of *Slc6a4*^*-/-*^ compared to WT mice one day after ONC, the significant genes (P < 0.05) which are known to regulate RGC death/survival and axon regeneration after ONC, are listed. (B) Enriched cellular processes and pathways in the VT *Slc6a4*^*-/-*^ retina compared to WT retina one day after ONC are shown. Bars indicate the degree of -log_10_ P value.
(TIF)

S6 Fig GPNMB expression in DBA/2J and DBA/2J-*Gpnmb*^*+*^ mice. GPNMB expression in RGCs are detected in DBA/2J-*Gpnmb*^*+*^ mice, but not DBA/2J mice. Scale bar represents 20 μm.
(TIF)

S7 Fig GPNMB promotes dorsal RGC survival and axon regeneration. (A) Schema of AAV2-Ctr or AAV2-GFP-*Gpnmb* virus infection in the dorsal retina of DBA/2J mice and RBPMS^+^ RGC survival analysis in the peripheral DN and DT retina two weeks after ONC. (B-C) In in DBA/2J mice, peripheral DN and DT RGCs are protective by AAV2-GFP-*Gpnmb* virus compared to AAV2-Ctr virus two weeks after ONC (n = 4–5 mice/condition, two-way ANOVA). (D) CTB-labeled regenerated axons beyond the injury site (*) are observed in DBA/2J mice after infection of AAV2-GFP-*Gpnmb* virus, but not AAV2-Ctr into the dorsal retina. (E-F) Quantitative analysis of axon regeneration in DBA/2J mice having AAV2-Ctr or AAV2-GFP-*Gpnmb* virus in the dorsal retina two weeks after ONC. The average of the distance of the CTB-labeled five longest axons from the lesion site *(E)* and the number of regenerated axons at 400 and 800 μm from the lesion site are shown *(F)*. (n = 4 mice/condition, two-tailed unpaired t-test (*E*), two-way ANOVA (*F*)). Data presented as mean ± SD. N.S., not significant; Scale bars represent 20 μm (B) and 100 μm (D).
(TIF)

S1 Table Genes significantly different in the VT retina between *Slc6a4*^*-/-*^ mutant and WT mice one day after optic nerve injury. Significant genes (P < 0.05) in the VT retina of *Slc6a4*^*-/-*^ compared to WT mice one day after ONC are listed. Gene expression levels in *Slc6a4*^*-/-*^ mutant are shown as Fold change of their expression over that in WT. Genes (in red) have been identified as genes differentially expressed in specific RGC subtypes after optic nerve injury [16].
(DOCX)

S1 Data Original Data sheets. (XLSX)
