# Supplementary figures and images for: Serotonin transporter-mediated molecular axis regulates regional retinal ganglion cell vulnerability and axon regeneration after nerve injury
Source: PLoS Genet. 2021 Nov 4;17(11):e1009885. doi: 10.1371/journal.pgen.1009885 (PMC8594818; doi:10.1371/journal.pgen.1009885)

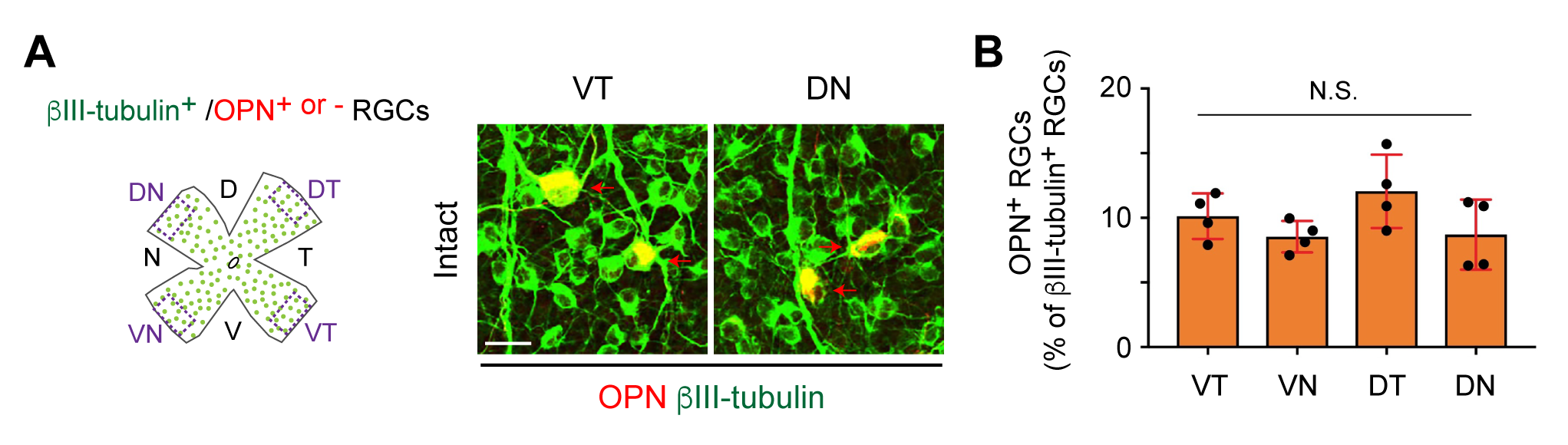

Supplement: S1 Fig — (A) Distribution of osteopontin (OPN)+ RGCs in four quadrants of the peripheral intact retina. Osteopontin (OPN)+/βIII-tubulin+ RGCs are detected in both VT and DN retina. (B) Quantitative analysis of osteopontin (OPN)+ RGCs (%) in four quadrants of the intact peripheral retina (n = 4, one-way ANOVA). Data presented as mean ± SD. N.S., not significant; Scale bar represents 20 μm. (TIF) [file pgen.1009885.s001.tif]

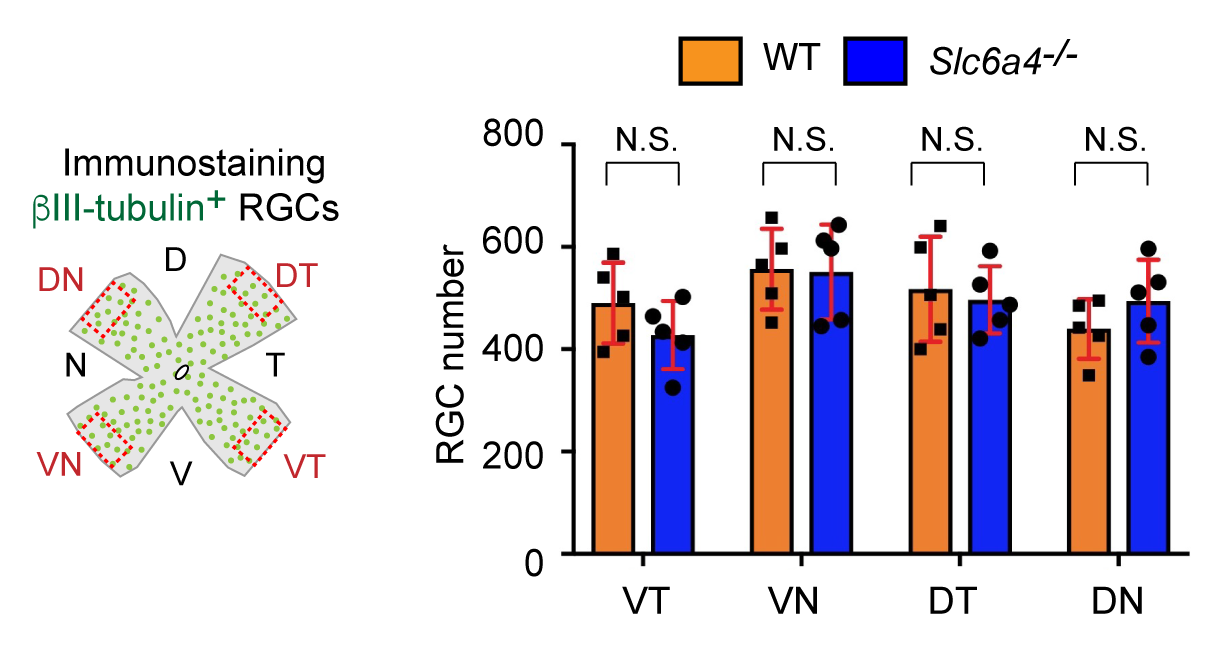

Supplement: S2 Fig — Quantification of βIII-tubulin+ RGCs in four quadrants of the intact peripheral retina in Slc6a4-/- and WT mice (n = 5/condition, two-way ANOVA). Data presented as mean ± SD; N.S., not significant. (TIF) [file pgen.1009885.s002.tif]

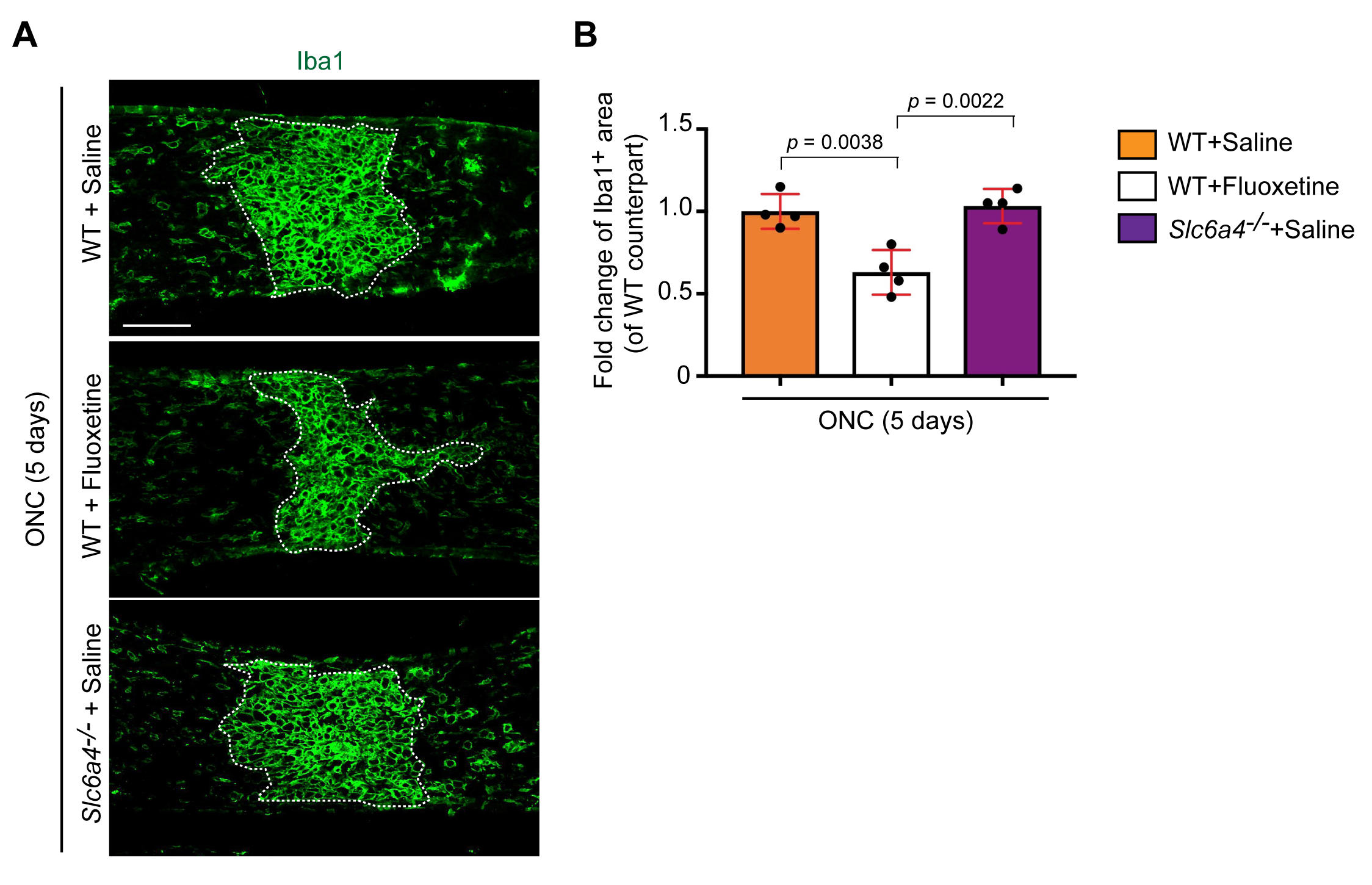

Supplement: S3 Fig — (A-B) Five days after ONC, WT mice treated with fluoxetine show a reduced area of Iba1+ activated microglia/macrophages at the lesion site in the optic nerve compared to WT or Slc6a4-/- mice with saline (n = 4/condition, one-way ANOVA). Data presented as mean ± SD. Scale bar represents 100 μm. (TIF) [file pgen.1009885.s003.tif]

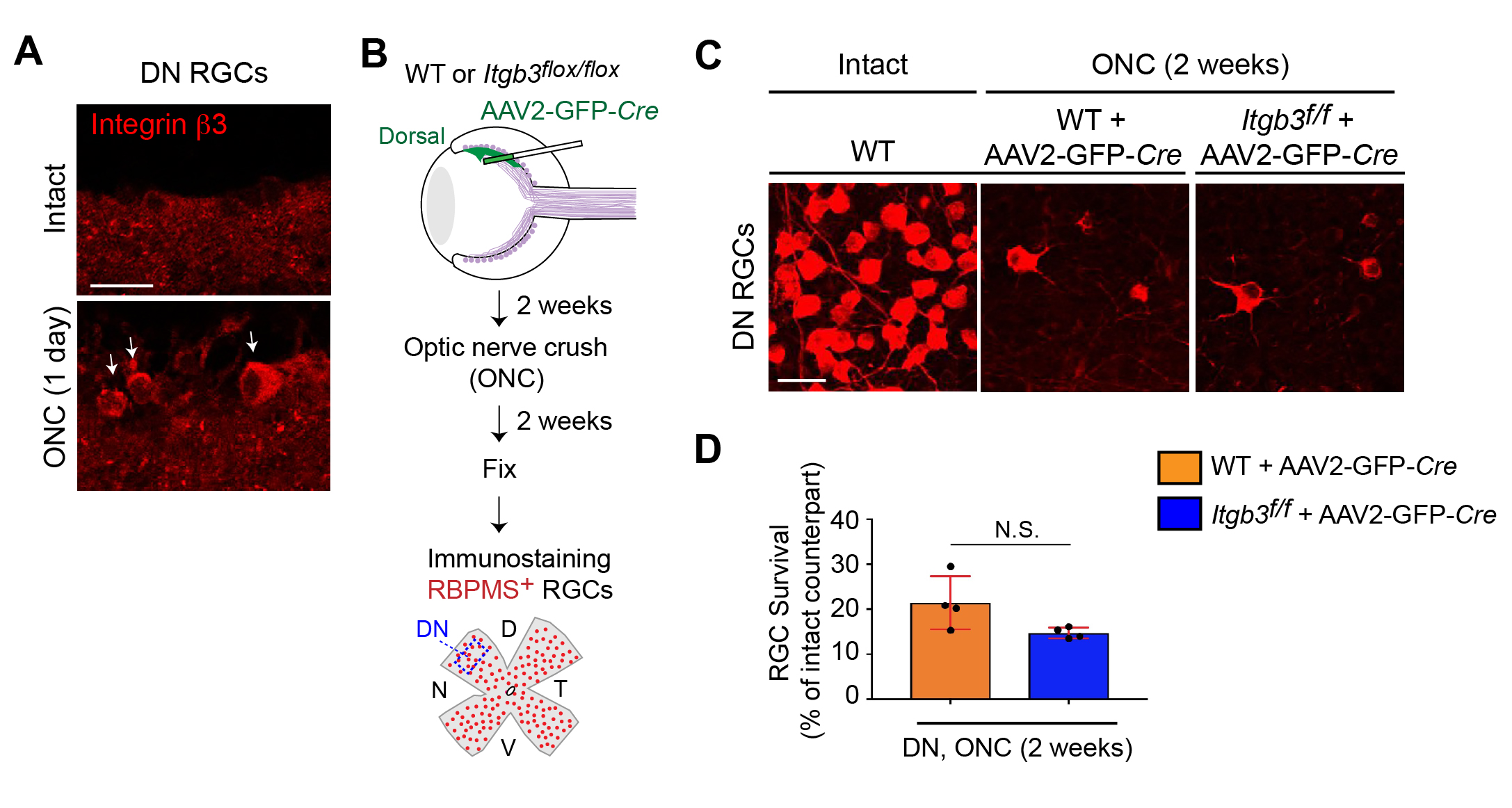

Supplement: S4 Fig — (A) One day after ONC, integrin β3 is upregulated in DN RGCs (arrows). (B) Schema of deletion of Itgb3 in the dorsal retina of Itgb3flox/flox mice after intravitreal injection of AAV2-GFP-Cre virus and RBPMS+ RGC survival analysis in the peripheral DN WT or Itgb3 flox/flox + AAV2-GFP-Cre retina two weeks after ONC. (C-D) DN RGCs in WT and Itgb3 flox/flox + AAV2-GFP-Cre retina are similarly vulnerable to injury (n = 4/condition, two-tailed unpaired t-test). Data presented as mean ± SD. N.S., not significant; Scale bars represent 20 μm. (JPG) [file pgen.1009885.s004.jpg]

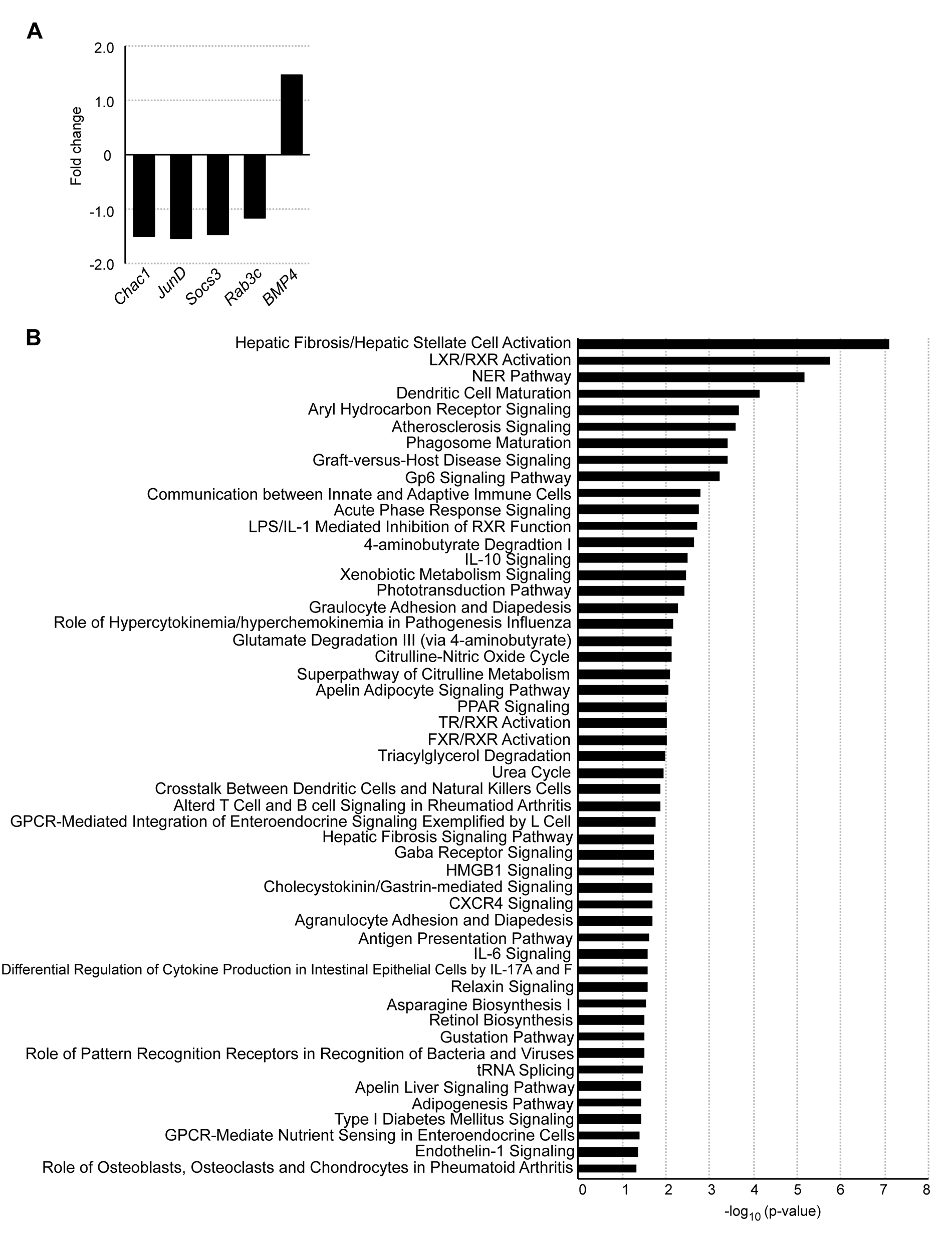

Supplement: S5 Fig — (A) In the VT retina of Slc6a4-/- compared to WT mice one day after ONC, the significant genes (P < 0.05) which are known to regulate RGC death/survival and axon regeneration after ONC, are listed. (B) Enriched cellular processes and pathways in the VT Slc6a4-/- retina compared to WT retina one day after ONC are shown. Bars indicate the degree of -log10 P value. (TIF) [file pgen.1009885.s005.tif]

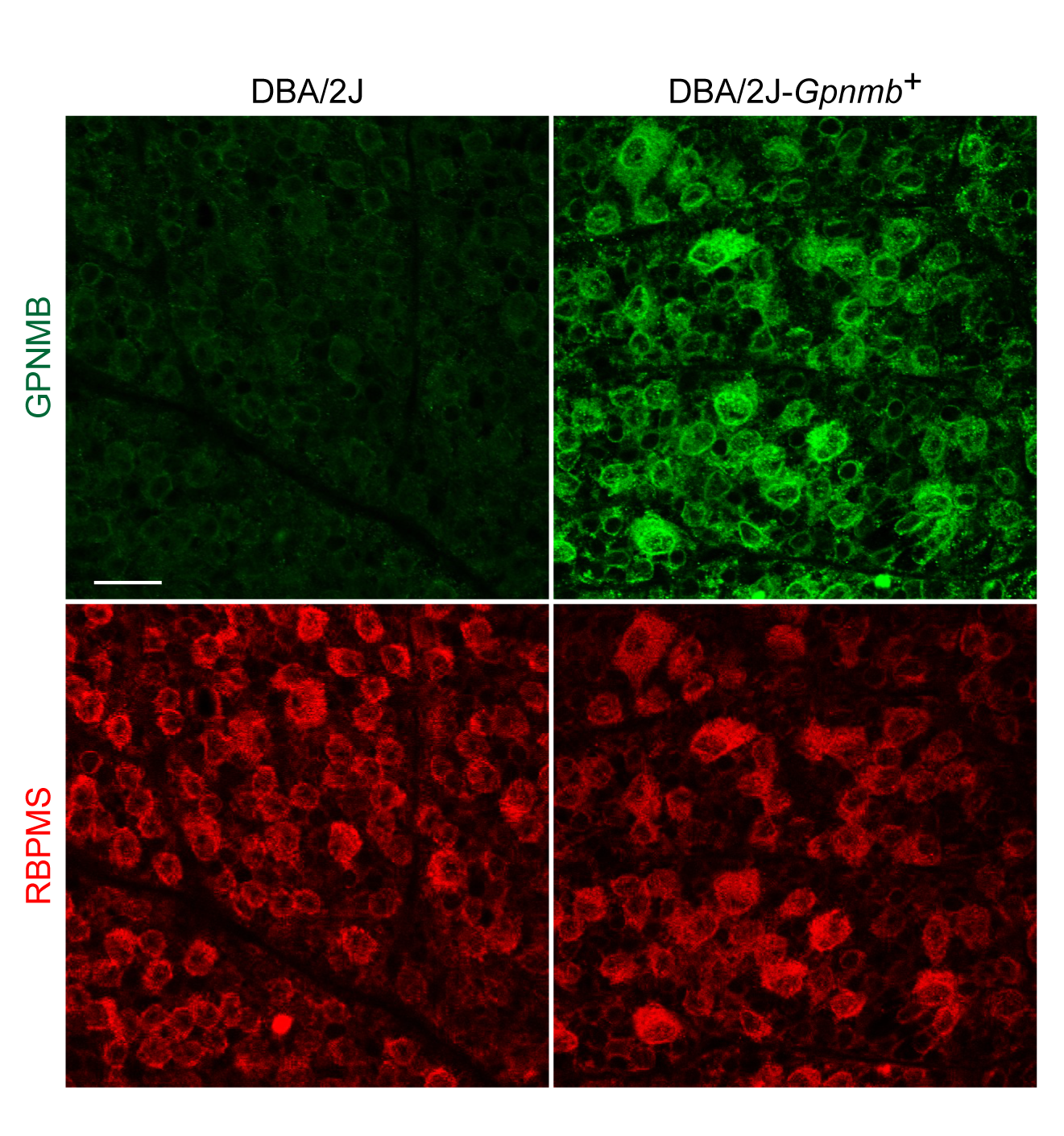

Supplement: S6 Fig — GPNMB expression in RGCs are detected in DBA/2J-Gpnmb+ mice, but not DBA/2J mice. Scale bar represents 20 μm. (TIF) [file pgen.1009885.s006.tif]

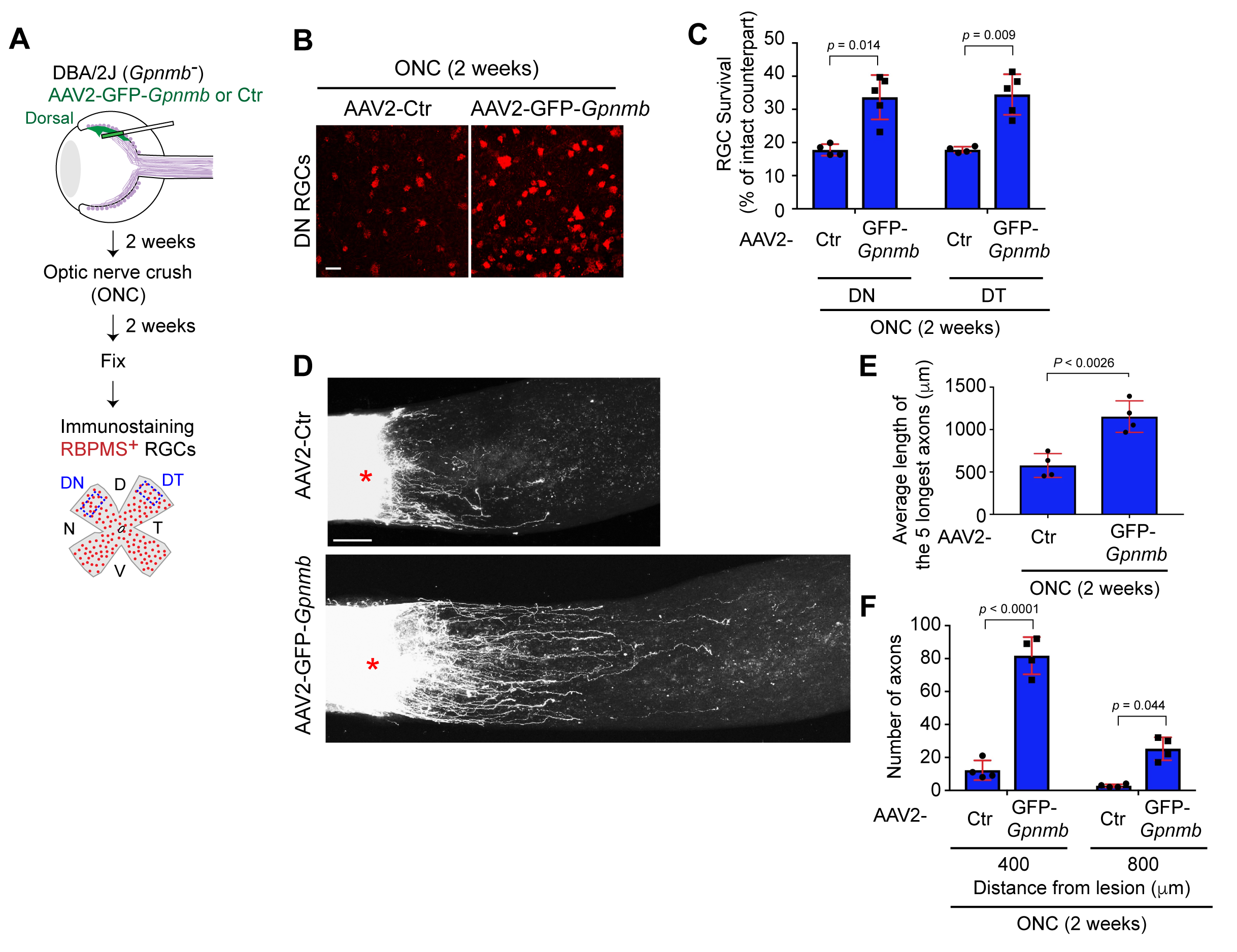

Supplement: S7 Fig — (A) Schema of AAV2-Ctr or AAV2-GFP-Gpnmb virus infection in the dorsal retina of DBA/2J mice and RBPMS+ RGC survival analysis in the peripheral DN and DT retina two weeks after ONC. (B-C) In in DBA/2J mice, peripheral DN and DT RGCs are protective by AAV2-GFP-Gpnmb virus compared to AAV2-Ctr virus two weeks after ONC (n = 4–5 mice/condition, two-way ANOVA). (D) CTB-labeled regenerated axons beyond the injury site (*) are observed in DBA/2J mice after infection of AAV2-GFP-Gpnmb virus, but not AAV2-Ctr into the dorsal retina. (E-F) Quantitative analysis of axon regeneration in DBA/2J mice having AAV2-Ctr or AAV2-GFP-Gpnmb virus in the dorsal retina two weeks after ONC. The average of the distance of the CTB-labeled five longest axons from the lesion site (E) and the number of regenerated axons at 400 and 800 μm from the lesion site are shown (F). (n = 4 mice/condition, two-tailed unpaired t-test (E), two-way ANOVA (F)). Data presented as mean ± SD. N.S., not significant; Scale bars represent 20 μm (B) and 100 μm (D). (TIF) [file pgen.1009885.s007.tif]
