# Supplementary material for: Serotonin transporter-mediated molecular axis regulates regional retinal ganglion cell vulnerability and axon regeneration after nerve injury
Source: PLoS Genet. 2021 Nov 4;17(11):e1009885. doi: 10.1371/journal.pgen.1009885 (PMC8594818; doi:10.1371/journal.pgen.1009885)
Supplement: S1 Table — Significant genes (P < 0.05) in the VT retina of Slc6a4-/- compared to WT mice one day after ONC are listed. Gene expression levels in Slc6a4-/- mutant are shown as Fold change of their expression over that in WT. Genes (in red) have been identified as genes differentially expressed in specific RGC subtypes after optic nerve injury [16]. (DOCX) [file pgen.1009885.s008.docx]

| **Name** | **Identifier** | **Fold change** |
| --- | --- | --- |
| Prss56 | ENSMUSG00000036480 | 3.69 |
| Gabra2 | ENSMUSG00000000560 | 1.95 |
| Eps8l1 | ENSMUSG00000006154 | -2.63 |
| Slc6a4 | ENSMUSG00000020838 | -1,532.11 |
| Srp54a | ENSMUSG00000073079 | -2.15 |
| Srp54b | ENSMUSG00000112449 | -655.61 |
| Slfn4 | ENSMUSG00000000204 | 11.85 |
| Wdfy1 | ENSMUSG00000073643 | 1.72 |
| Dynlt1b | ENSMUSG00000096255 | 2.08 |
| Gm3435 | ENSMUSG00000116895 | 2.16 |
| Cdcp3 | ENSMUSG00000006204 | 1.53 |
| Crb1 | ENSMUSG00000063681 | -2.04 |
| Opn1sw | ENSMUSG00000058831 | -2.42 |
| Nell1 | ENSMUSG00000055409 | -1.8 |
| Hist1h2bq | ENSMUSG00000069307 | -2.77 |
| Gm21541 | ENSMUSG00000094065 | 17.07 |
| Hspa1b | ENSMUSG00000090877 | -1.43 |
| Tnfrsf8 | ENSMUSG00000028602 | -1.72 |
| Steap2 | ENSMUSG00000015653 | 1.41 |
| Gpnmb | ENSMUSG00000029816 | 1.98 |
| Galnt2 | ENSMUSG00000089704 | -1.72 |
| Irf3 | ENSMUSG00000003184 | 1.42 |
| H1f2 | ENSMUSG00000036181 | -1.39 |
| Sema7a | ENSMUSG00000038264 | -1.41 |
| H2ac19 | ENSMUSG00000063954 | -1.85 |
| Col1a1 | ENSMUSG00000001506 | -2.55 |
| Cyp4f15 | ENSMUSG00000073424 | 3.1 |
| Ptp4a1_1 | ENSMUSG00000026064 | -2.04 |
| Gfap | ENSMUSG00000020932 | -1.36 |
| Rec8 | ENSMUSG00000002324 | 1.63 |
| Zfp541 | ENSMUSG00000078796 | 1.99 |
| Aldh1a1 | ENSMUSG00000053279 | 1.67 |
| H4c8 | ENSMUSG00000060981 | -1.73 |
| Pmel | ENSMUSG00000025359 | 1.93 |
| Igfbp5 | ENSMUSG00000026185 | 1.34 |
| Tns1 | ENSMUSG00000055322 | 1.34 |
| Ism2 | ENSMUSG00000050671 | 2.24 |
| Gsta3 | ENSMUSG00000025934 | 2.26 |
| Gm20388 | ENSMUSG00000092329 | 1.46 |
| Hspa1a | ENSMUSG00000091971 | -1.32 |
| Asns | ENSMUSG00000029752 | -1.33 |
| Ctsh | ENSMUSG00000032359 | 1.58 |
| Pcdha8 | ENSMUSG00000103800 | -1.52 |
| Cdkn1a | ENSMUSG00000023067 | -2.38 |
| Bub3 | ENSMUSG00000066979 | 1.31 |
| Bpifb9b | ENSMUSG00000067996 | -3.59 |
| Jund | ENSMUSG00000071076 | -1.53 |
| Hist1h4m | ENSMUSG00000069306 | -1.73 |
| Ltbp1 | ENSMUSG00000001870 | 1.75 |
| Gm14744 | ENSMUSG00000079522 | -3.31 |
| Tmem181a | ENSMUSG00000038141 | 1.3 |
| Rapsn | ENSMUSG00000002104 | 2.56 |
| Folr1 | ENSMUSG00000001827 | 1.55 |
| Vax2 | ENSMUSG00000034777 | -1.83 |
| Dct | ENSMUSG00000022129 | 2 |
| Col26a1 | ENSMUSG00000004415 | -2.01 |
| Slc4a4 | ENSMUSG00000060961 | 1.32 |
| Pde11a | ENSMUSG00000075270 | -1.59 |
| Cgnl1 | ENSMUSG00000032232 | 1.31 |
| Tgtp1 | ENSMUSG00000078922 | 4.35 |
| H3c6 | ENSMUSG00000069273 | -1.49 |
| Clec18a | ENSMUSG00000033633 | 1.95 |
| H4c2 | ENSMUSG00000069266 | -1.61 |
| Ina | ENSMUSG00000034336 | -1.3 |
| Duxbl2 | ENSMUSG00000072675 | -1.82 |
| Pde6c | ENSMUSG00000024992 | -1.18 |
| Ak4 | ENSMUSG00000028527 | 1.19 |
| Slc26a7 | ENSMUSG00000040569 | 1.65 |
| Serpine1 | ENSMUSG00000037411 | 2.5 |
| Tprkb | ENSMUSG00000054226 | 1.29 |
| Rsad2 | ENSMUSG00000020641 | 2.1 |
| Acaa2 | ENSMUSG00000036880 | 1.32 |
| Col11a1 | ENSMUSG00000027966 | 1.36 |
| Bpifa1 | ENSMUSG00000027483 | -3.27 |
| Npcd | ENSMUSG00000089837 | 13.28 |
| Vps53 | ENSMUSG00000017288 | 1.19 |
| Cbarp | ENSMUSG00000035640 | -1.29 |
| Slc15a2 | ENSMUSG00000022899 | -1.6 |
| Gm28539 | ENSMUSG00000099908 | -10.01 |
| Pld5 | ENSMUSG00000055214 | 1.71 |
| Gbp3 | ENSMUSG00000028268 | 1.78 |
| Gm14743 | ENSMUSG00000079519 | -12.78 |
| H3c4 | ENSMUSG00000099583 | -1.8 |
| Onecut2 | ENSMUSG00000045991 | -1.29 |
| Iqgap2 | ENSMUSG00000021676 | 1.57 |
| B2m | ENSMUSG00000060802 | 1.46 |
| Ifit3 | ENSMUSG00000074896 | 1.47 |
| Samd9l | ENSMUSG00000047735 | 1.44 |
| mt-Nd3 | ENSMUSG00000064360 | 1.39 |
| Obp1a | ENSMUSG00000067684 | -3.69 |
| Insm1 | ENSMUSG00000068154 | -1.43 |
| H2ac18 | ENSMUSG00000064220 | -1.68 |
| Lcn2 | ENSMUSG00000026822 | 1.81 |
| H2ac7 | ENSMUSG00000071478 | -2.34 |
| Pcdha11_1 | ENSMUSG00000007440 | -1.29 |
| Ociad2 | ENSMUSG00000029153 | 1.56 |
| H4c11 | ENSMUSG00000067455 | -1.73 |
| A2m | ENSMUSG00000030111 | 1.53 |
| Trpc6 | ENSMUSG00000031997 | -1.28 |
| Ifitm3 | ENSMUSG00000025492 | 1.75 |
| Obp1b | ENSMUSG00000067679 | -2.93 |
| Ctsl | ENSMUSG00000021477 | 1.17 |
| Opn1mw | ENSMUSG00000031394 | 1.27 |
| Vcan | ENSMUSG00000021614 | 1.86 |
| Vmn2r7 | ENSMUSG00000062200 | 4.5 |
| Mill2 | ENSMUSG00000040987 | 2.84 |
| Rarres2 | ENSMUSG00000009281 | 1.82 |
| Gm13304 | ENSMUSG00000073878 | -6 |
| Morn5 | ENSMUSG00000026894 | 5.99 |
| Clec14a | ENSMUSG00000045930 | 1.68 |
| Tyrp1 | ENSMUSG00000005994 | 1.78 |
| Ahnak2 | ENSMUSG00000072812 | -1.44 |
| Wfdc1 | ENSMUSG00000023336 | 1.78 |
| Fbln2 | ENSMUSG00000064080 | 2.08 |
| Lgals3bp | ENSMUSG00000033880 | 1.45 |
| Gsn | ENSMUSG00000026879 | 1.47 |
| Lfng | ENSMUSG00000029570 | -1.41 |
| Gemin4 | ENSMUSG00000049396 | 1.97 |
| H4c1 | ENSMUSG00000060093 | -1.46 |
| Akr1c14 | ENSMUSG00000033715 | 2.31 |
| Ccdc142 | ENSMUSG00000107499 | -2.54 |
| Crhbp | ENSMUSG00000021680 | 1.96 |
| H3c1 | ENSMUSG00000069265 | -1.53 |
| Slc6a20a | ENSMUSG00000036814 | 1.55 |
| Suclg2 | ENSMUSG00000061838 | 1.43 |
| Trim67 | ENSMUSG00000036913 | -1.3 |
| Muc2 | ENSMUSG00000025515 | 1.65 |
| H1f4 | ENSMUSG00000051627 | -1.44 |
| Capn3 | ENSMUSG00000079110 | 2.77 |
| Adh1 | ENSMUSG00000074207 | 4.38 |
| Vip | ENSMUSG00000019772 | 1.37 |
| Pcdha6 | ENSMUSG00000103707 | -1.39 |
| Gm28035 | ENSMUSG00000099041 | -1.32 |
| H3c7 | ENSMUSG00000100210 | -1.43 |
| Pcdhb7 | ENSMUSG00000045062 | -1.34 |
| Tpm3 | ENSMUSG00000027940 | -1.17 |
| Mgst1 | ENSMUSG00000008540 | 1.65 |
| Arhgap29 | ENSMUSG00000039831 | 1.27 |
| Atp1b3 | ENSMUSG00000032412 | 1.38 |
| Gm20075 | ENSMUSG00000114133 | -2.33 |
| Ppm1n | ENSMUSG00000030402 | -1.31 |
| Slco1c1 | ENSMUSG00000030235 | 1.52 |
| Fam107a | ENSMUSG00000021750 | 1.45 |
| Gm43302 | ENSMUSG00000079362 | -167.52 |
| Dzip1 | ENSMUSG00000042156 | -1.25 |
| Alpk2 | ENSMUSG00000032845 | 1.65 |
| Ifi27 | ENSMUSG00000064215 | 1.48 |
| Ces1d | ENSMUSG00000056973 | 2.18 |
| Xlr3b | ENSMUSG00000073125 | -1.65 |
| Slc13a4 | ENSMUSG00000029843 | 1.92 |
| Sgsm2 | ENSMUSG00000038351 | -1.26 |
| S100a8 | ENSMUSG00000056054 | 4.07 |
| Slc16a12 | ENSMUSG00000009378 | 1.85 |
| Rab3c | ENSMUSG00000021700 | -1.16 |
| Oas2 | ENSMUSG00000032690 | 5.61 |
| Lrrc4b | ENSMUSG00000047085 | -1.26 |
| A730049H05Rik | ENSMUSG00000048636 | 2.47 |
| Serpina3n | ENSMUSG00000021091 | 1.81 |
| Mlana | ENSMUSG00000024806 | 2.43 |
| Cebpd | ENSMUSG00000071637 | -1.48 |
| Cavin2 | ENSMUSG00000045954 | 1.62 |
| Slc22a28 | ENSMUSG00000063590 | 2.04 |
| H4c3 | ENSMUSG00000060678 | -1.38 |
| Nxph4 | ENSMUSG00000040258 | -1.83 |
| Dlgap3 | ENSMUSG00000042388 | -1.39 |
| F5 | ENSMUSG00000026579 | 2.05 |
| Penk | ENSMUSG00000045573 | 2.22 |
| Ccnb1ip1 | ENSMUSG00000071470 | -1.39 |
| Sult1a1 | ENSMUSG00000030711 | 1.57 |
| Gstm2 | ENSMUSG00000040562 | 2.1 |
| Samd14 | ENSMUSG00000047181 | -1.16 |
| Aox1 | ENSMUSG00000063558 | 1.55 |
| Igflr1 | ENSMUSG00000036826 | 4.53 |
| Igfbp4 | ENSMUSG00000017493 | 1.31 |
| Otop3 | ENSMUSG00000018862 | -1.4 |
| Scel | ENSMUSG00000022123 | 1.85 |
| 2210418O10Rik | ENSMUSG00000078894 | 1.4 |
| Ifi27l2a | ENSMUSG00000079017 | 3.24 |
| Pcdhb17 | ENSMUSG00000046387 | -1.16 |
| Igf2bp1 | ENSMUSG00000013415 | 2.47 |
| Atp1a2 | ENSMUSG00000007097 | 1.86 |
| Slc7a11 | ENSMUSG00000027737 | 1.48 |
| Ecrg4 | ENSMUSG00000026051 | 2.34 |
| Ngfr | ENSMUSG00000000120 | -1.25 |
| H4c9 | ENSMUSG00000060639 | -1.39 |
| Zdbf2 | ENSMUSG00000027520 | -1.24 |
| Nat8f6 | ENSMUSG00000079495 | 4.82 |
| Enox1 | ENSMUSG00000022012 | -1.33 |
| Itga6 | ENSMUSG00000027111 | -1.37 |
| Slc26a4 | ENSMUSG00000020651 | 2.31 |
| Iqcn | ENSMUSG00000110622 | -1.88 |
| Kctd8 | ENSMUSG00000037653 | -1.24 |
| Cpq | ENSMUSG00000039007 | 1.34 |
| Nradd | ENSMUSG00000032491 | 1.78 |
| Gldc | ENSMUSG00000024827 | 1.36 |
| Tmprss11e | ENSMUSG00000054537 | 2.16 |
| Muc5b | ENSMUSG00000066108 | -3.08 |
| Efemp1 | ENSMUSG00000020467 | 1.43 |
| Flna | ENSMUSG00000031328 | 1.22 |
| Rbm42 | ENSMUSG00000036733 | -1.23 |
| Magi1 | ENSMUSG00000045095 | -1.15 |
| Hr | ENSMUSG00000022096 | -1.23 |
| Tpgs1 | ENSMUSG00000020308 | -1.6 |
| Tmem151b | ENSMUSG00000096847 | -1.36 |
| Cdk5r2 | ENSMUSG00000090071 | -1.34 |
| Col9a1 | ENSMUSG00000026147 | 1.22 |
| Nap1l2 | ENSMUSG00000082229 | -1.24 |
| Best2 | ENSMUSG00000052819 | 2.11 |
| Slc38a8 | ENSMUSG00000034224 | 2.92 |
| Chrdl1 | ENSMUSG00000031283 | 1.23 |
| Map1s | ENSMUSG00000019261 | -1.27 |
| Mylk | ENSMUSG00000022836 | -1.22 |
| Wdr66 | ENSMUSG00000029442 | -1.24 |
| Gja1 | ENSMUSG00000050953 | 1.89 |
| Pcsk1n | ENSMUSG00000039278 | -1.6 |
| Tac1 | ENSMUSG00000061762 | 1.38 |
| Gngt1 | ENSMUSG00000029663 | 1.22 |
| Tmsb4x | ENSMUSG00000049775 | 1.22 |
| Ptgds | ENSMUSG00000015090 | 1.71 |
| Wls | ENSMUSG00000028173 | 1.41 |
| Igf2bp3 | ENSMUSG00000029814 | -2.91 |
| Il1rn | ENSMUSG00000026981 | 3.06 |
| Necab2 | ENSMUSG00000031837 | 1.33 |
| Ogn | ENSMUSG00000021390 | 1.82 |
| Itpr3 | ENSMUSG00000042644 | 1.44 |
| Lyz2 | ENSMUSG00000069516 | 1.68 |
| Slc1a4 | ENSMUSG00000020142 | -1.31 |
| Slc19a1 | ENSMUSG00000001436 | 1.23 |
| Lrat | ENSMUSG00000028003 | 1.38 |
| Zfp185 | ENSMUSG00000031351 | 1.59 |
| Scand1 | ENSMUSG00000046229 | -1.93 |
| Obp2a | ENSMUSG00000062061 | -2.94 |
| Hs3st3b1 | ENSMUSG00000070407 | -1.14 |
| Srp54c | ENSMUSG00000079108 | -1.21 |
| Slc2a1 | ENSMUSG00000028645 | 1.14 |
| Ucp2 | ENSMUSG00000033685 | 1.4 |
| Tbx5 | ENSMUSG00000018263 | 2.89 |
| C2 | ENSMUSG00000024371 | 1.86 |
| Mfrp | ENSMUSG00000034739 | 1.6 |
| Fscn2 | ENSMUSG00000025380 | -1.21 |
| Col6a1 | ENSMUSG00000001119 | 1.26 |
| Pcdh8 | ENSMUSG00000036422 | -1.34 |
| Tns3 | ENSMUSG00000020422 | 1.21 |
| H4c12 | ENSMUSG00000064288 | -1.57 |
| Cdkn2c | ENSMUSG00000028551 | 1.29 |
| Bambi | ENSMUSG00000024232 | 1.41 |
| Ctsa | ENSMUSG00000017760 | 1.14 |
| Mme | ENSMUSG00000027820 | 1.74 |
| Gbp9 | ENSMUSG00000029298 | 1.49 |
| Clip2 | ENSMUSG00000063146 | -1.22 |
| Scn4a | ENSMUSG00000001027 | -1.27 |
| Atp13a4 | ENSMUSG00000038094 | 2.85 |
| Pcdh9 | ENSMUSG00000055421 | -1.13 |
| Nat8f4 | ENSMUSG00000068299 | 1.29 |
| Emp3 | ENSMUSG00000040212 | 1.99 |
| Frk | ENSMUSG00000019779 | 1.74 |
| Atp5md | ENSMUSG00000071528 | 1.22 |
| Dse | ENSMUSG00000039497 | 1.54 |
| Duxbl3 | ENSMUSG00000072672 | -2.01 |
| Hax1 | ENSMUSG00000027944 | 1.23 |
| Bpifb9a | ENSMUSG00000067998 | -4.33 |
| Malt1 | ENSMUSG00000032688 | 1.22 |
| Mdfic | ENSMUSG00000041390 | 1.52 |
| Arhgap31 | ENSMUSG00000022799 | -1.21 |
| Slc43a2 | ENSMUSG00000038178 | -1.26 |
| Pear1 | ENSMUSG00000028073 | 1.72 |
| Olfml2a | ENSMUSG00000046618 | 1.68 |
| Kcnj13 | ENSMUSG00000079436 | 1.58 |
| H3c15 | ENSMUSG00000081058 | -1.41 |
| Sema3c | ENSMUSG00000028780 | 1.35 |
| Wdr62 | ENSMUSG00000037020 | 2.12 |
| Cntn3 | ENSMUSG00000030075 | 1.49 |
| S100a9 | ENSMUSG00000056071 | 2.18 |
| Islr | ENSMUSG00000037206 | 1.8 |
| Kcnh7 | ENSMUSG00000059742 | -1.21 |
| Rasl10b | ENSMUSG00000020684 | -1.21 |
| Slc2a13 | ENSMUSG00000036298 | -1.14 |
| Ass1 | ENSMUSG00000076441 | 1.22 |
| Colec12 | ENSMUSG00000036103 | 1.86 |
| Gm49909 | ENSMUSG00000117098 | -1.46 |
| Notum | ENSMUSG00000042988 | 2.07 |
| Cerk | ENSMUSG00000035891 | -1.13 |
| Arhgap28 | ENSMUSG00000024043 | -1.31 |
| Mybl1 | ENSMUSG00000025912 | 1.3 |
| Acacb | ENSMUSG00000042010 | 1.49 |
| H2ac15 | ENSMUSG00000063021 | -2.05 |
| Atp10d | ENSMUSG00000046808 | 1.38 |
| Fhdc1 | ENSMUSG00000041842 | 1.39 |
| Ugt3a2 | ENSMUSG00000049152 | 5.32 |
| Kdm6a | ENSMUSG00000037369 | -1.3 |
| Slc16a4 | ENSMUSG00000027896 | 1.69 |
| Gm4737 | ENSMUSG00000048087 | 7.17 |
| Cd24a | ENSMUSG00000047139 | -1.34 |
| Gm3448 | ENSMUSG00000079710 | 1.27 |
| Cited2 | ENSMUSG00000039910 | -1.3 |
| Mfap4 | ENSMUSG00000042436 | 1.88 |
| Oas1b | ENSMUSG00000029605 | 2.12 |
| H4c6 | ENSMUSG00000069274 | -1.36 |
| Gbp6 | ENSMUSG00000104713 | 1.32 |
| Ccdc85b | ENSMUSG00000095098 | -1.31 |
| Pdyn | ENSMUSG00000027400 | 1.29 |
| Ebf2 | ENSMUSG00000022053 | -2.65 |
| Pcdhgb2 | ENSMUSG00000102748 | -1.21 |
| Dnase1l1 | ENSMUSG00000019088 | 1.84 |
| Bex1 | ENSMUSG00000050071 | -1.23 |
| Pde1c | ENSMUSG00000004347 | 1.13 |
| Thra | ENSMUSG00000058756 | -1.13 |
| Tril | ENSMUSG00000043496 | -1.28 |
| Gsta1 | ENSMUSG00000074183 | 89.63 |
| Cldn1 | ENSMUSG00000022512 | 1.47 |
| Serinc4 | ENSMUSG00000046110 | 1.14 |
| Etfb | ENSMUSG00000004610 | 1.32 |
| 4932438H23Rik | ENSMUSG00000039851 | 2.65 |
| Prdm16 | ENSMUSG00000039410 | 1.52 |
| Fgfr2 | ENSMUSG00000030849 | 1.54 |
| Fcgrt | ENSMUSG00000003420 | 1.4 |
| Adrb2 | ENSMUSG00000045730 | 1.66 |
| Plin4 | ENSMUSG00000002831 | 1.62 |
| Eif2ak2 | ENSMUSG00000024079 | 1.36 |
| Fam177a | ENSMUSG00000095595 | -6.55 |
| Col18a1 | ENSMUSG00000001435 | 1.48 |
| Cit | ENSMUSG00000029516 | -1.22 |
| Elfn2 | ENSMUSG00000043460 | -1.2 |
| Slc25a20 | ENSMUSG00000032602 | 1.28 |
| Myt1l | ENSMUSG00000061911 | -1.2 |
| Gstm1 | ENSMUSG00000058135 | 1.37 |
| H2-M3 | ENSMUSG00000016206 | 1.84 |
| Bcap29 | ENSMUSG00000020650 | 1.25 |
| Emilin2 | ENSMUSG00000024053 | 2.03 |
| Gm4631 | ENSMUSG00000078899 | -1.2 |
| Pcdh11x | ENSMUSG00000034755 | -1.21 |
| Has2 | ENSMUSG00000022367 | 2.33 |
| Mc4r | ENSMUSG00000047259 | -1.81 |
| mt-Co3 | ENSMUSG00000064358 | 1.19 |
| Abi3bp | ENSMUSG00000035258 | 1.48 |
| Tmem184a | ENSMUSG00000036687 | 3.08 |
| Elmo3 | ENSMUSG00000014791 | 1.33 |
| Sptb | ENSMUSG00000021061 | -1.13 |
| Kmo | ENSMUSG00000039783 | 8.65 |
| Pcdhga3 | ENSMUSG00000104346 | -1.2 |
| Ifi204 | ENSMUSG00000073489 | 2.37 |
| Zfp385b | ENSMUSG00000027016 | -1.2 |
| Stx1b | ENSMUSG00000030806 | -1.13 |
| Arhgap15 | ENSMUSG00000049744 | -1.36 |
| Ltbp2 | ENSMUSG00000002020 | 1.66 |
| Cox7c | ENSMUSG00000017778 | 1.2 |
| H2-Aa | ENSMUSG00000036594 | 2 |
| Dkk3 | ENSMUSG00000030772 | 1.19 |
| Shisa8 | ENSMUSG00000096883 | -1.38 |
| Dlgap4 | ENSMUSG00000061689 | -1.2 |
| Aifm3 | ENSMUSG00000022763 | 1.46 |
| Scnm1 | ENSMUSG00000092607 | 1.3 |
| Ifit1 | ENSMUSG00000034459 | 1.62 |
| Zfp579 | ENSMUSG00000051550 | -1.42 |
| Gm37389 | ENSMUSG00000103124 | 2.78 |
| Gng11 | ENSMUSG00000032766 | 1.41 |
| Timp3 | ENSMUSG00000020044 | 1.3 |
| Fam129a | ENSMUSG00000026483 | 1.62 |
| Shc3 | ENSMUSG00000021448 | -1.27 |
| Adrb1 | ENSMUSG00000035283 | -1.23 |
| Gse1 | ENSMUSG00000031822 | -1.19 |
| Dapl1 | ENSMUSG00000026989 | 1.32 |
| Ppia | ENSMUSG00000071866 | 1.19 |
| Smim1 | ENSMUSG00000078350 | 1.25 |
| Mecom | ENSMUSG00000027684 | 1.57 |
| Hgsnat | ENSMUSG00000037260 | 1.13 |
| Morc2b | ENSMUSG00000048602 | -1.63 |
| Slc6a13 | ENSMUSG00000030108 | 1.8 |
| Crocc | ENSMUSG00000040860 | -1.19 |
| Bcl3 | ENSMUSG00000053175 | -2.54 |
| Gfpt2 | ENSMUSG00000020363 | 1.8 |
| Gpx8 | ENSMUSG00000021760 | 1.31 |
| Ppl | ENSMUSG00000039457 | 1.51 |
| Meltf | ENSMUSG00000022780 | -2.75 |
| Ctsd | ENSMUSG00000007891 | 1.19 |
| Hcls1 | ENSMUSG00000022831 | -1.35 |
| Fam57a | ENSMUSG00000069808 | 1.39 |
| Rbp1 | ENSMUSG00000046402 | 1.32 |
| Krt23 | ENSMUSG00000006777 | -3.21 |
| Lgals1 | ENSMUSG00000068220 | 1.34 |
| Dcx | ENSMUSG00000031285 | -1.27 |
| Lrch4 | ENSMUSG00000093445 | 1.78 |
| Gstm4 | ENSMUSG00000027890 | 1.65 |
| Mllt11 | ENSMUSG00000053192 | 1.19 |
| Cacng4 | ENSMUSG00000020723 | -1.22 |
| Slc32a1 | ENSMUSG00000037771 | -1.2 |
| Cdh3 | ENSMUSG00000061048 | 1.51 |
| Ano3 | ENSMUSG00000074968 | -1.2 |
| Prtg | ENSMUSG00000036030 | -1.26 |
| Col9a3 | ENSMUSG00000027570 | 1.37 |
| Hsd17b11 | ENSMUSG00000029311 | 1.4 |
| P2ry1 | ENSMUSG00000027765 | 1.27 |
| Ndufa3 | ENSMUSG00000035674 | 1.21 |
| Il1b | ENSMUSG00000027398 | 2.23 |
| Lmf1 | ENSMUSG00000002279 | 1.28 |
| Minar2 | ENSMUSG00000050875 | 1.2 |
| Rhob | ENSMUSG00000054364 | -1.2 |
| H2ac6 | ENSMUSG00000069270 | -1.32 |
| Dsg1a | ENSMUSG00000069441 | 2.37 |
| Llgl2 | ENSMUSG00000020782 | -1.18 |
| Slc7a5 | ENSMUSG00000040010 | -1.25 |
| mt-Atp6 | ENSMUSG00000064357 | 1.18 |
| Bin2 | ENSMUSG00000098112 | -1.74 |
| Optc | ENSMUSG00000010311 | 1.75 |
| Nkd2 | ENSMUSG00000021567 | 1.74 |
| Apoc1 | ENSMUSG00000040564 | 2.93 |
| Gstt1 | ENSMUSG00000001663 | 1.31 |
| Cacna1h | ENSMUSG00000024112 | -1.18 |
| Gm13889 | ENSMUSG00000087006 | -1.36 |
| Isyna1 | ENSMUSG00000019139 | 1.27 |
| Mier2 | ENSMUSG00000042570 | -1.22 |
| Bin1 | ENSMUSG00000024381 | -1.12 |
| Lgi4 | ENSMUSG00000036560 | -1.33 |
| H3c14 | ENSMUSG00000093769 | -1.33 |
| Syn1 | ENSMUSG00000037217 | -1.22 |
| Tprn | ENSMUSG00000048707 | 1.37 |
| Nat8f5 | ENSMUSG00000079494 | 2.19 |
| Grm6 | ENSMUSG00000000617 | -1.18 |
| Ccdc148 | ENSMUSG00000036641 | 1.36 |
| Gdpd5 | ENSMUSG00000035314 | -1.12 |
| Tspyl4 | ENSMUSG00000039485 | -1.18 |
| Ephx1 | ENSMUSG00000038776 | 1.32 |
| Eif2s3x | ENSMUSG00000035150 | -1.18 |
| Vbp1 | ENSMUSG00000031197 | 1.12 |
| Ifitm2 | ENSMUSG00000060591 | 1.5 |
| Fam13a | ENSMUSG00000037709 | 1.2 |
| Tbcc | ENSMUSG00000036430 | -1.19 |
| Vstm2l | ENSMUSG00000037843 | -1.29 |
| Pcdhb16 | ENSMUSG00000047910 | -1.12 |
| Zfp69 | ENSMUSG00000064141 | -2.07 |
| Gnaz | ENSMUSG00000040009 | -1.12 |
| Tnfsf13 | ENSMUSG00000089669 | 1.19 |
| Gm45861 | ENSMUSG00000110333 | -1.72 |
| Heca | ENSMUSG00000039879 | -1.12 |
| Gm26992 | ENSMUSG00000098078 | -4.22 |
| Plec | ENSMUSG00000022565 | -1.12 |
| Col6a6 | ENSMUSG00000043719 | 1.64 |
| Apoa2 | ENSMUSG00000005681 | 2.73 |
| Ccr1 | ENSMUSG00000025804 | 2.05 |
| Ak5 | ENSMUSG00000039058 | 1.45 |
| Zfp804b | ENSMUSG00000092094 | -1.19 |
| Ccnd2 | ENSMUSG00000000184 | 1.59 |
| Creb5 | ENSMUSG00000053007 | -1.39 |
| Gng10 | ENSMUSG00000038607 | 1.28 |
| Kyat1 | ENSMUSG00000039648 | 1.22 |
| H2-K1 | ENSMUSG00000061232 | 1.27 |
| Socs3 | ENSMUSG00000053113 | -1.46 |
| Pon3 | ENSMUSG00000029759 | 1.65 |
| Matn2 | ENSMUSG00000022324 | 1.6 |
| Pnpla7 | ENSMUSG00000036833 | 1.25 |
| Ccdc89 | ENSMUSG00000044362 | 1.33 |
| Prps2 | ENSMUSG00000025742 | 1.27 |
| Cyp4f14 | ENSMUSG00000024292 | -1.58 |
| Scml4 | ENSMUSG00000044770 | -1.86 |
| Helz2 | ENSMUSG00000027580 | 1.33 |
| Serpine3 | ENSMUSG00000091155 | 2.18 |
| Krt6a | ENSMUSG00000058354 | -2.88 |
| Gm14327 | ENSMUSG00000074521 | 1.42 |
| Gm4724 | ENSMUSG00000078897 | 1.9 |
| Tdrd9 | ENSMUSG00000054003 | 1.19 |
| Itih4 | ENSMUSG00000021922 | 2.95 |
| Nxn | ENSMUSG00000020844 | 1.21 |
| Igf2bp2 | ENSMUSG00000033581 | 1.51 |
| Usp2 | ENSMUSG00000032010 | -1.17 |
| Edn3 | ENSMUSG00000027524 | 1.61 |
| Rims4 | ENSMUSG00000035226 | -1.19 |
| Gldn | ENSMUSG00000046167 | -1.6 |
| Fdx2 | ENSMUSG00000079677 | -1.51 |
| Acod1 | ENSMUSG00000022126 | 2.73 |
| Fzd6 | ENSMUSG00000022297 | 1.29 |
| Rhou | ENSMUSG00000039960 | -1.2 |
| A730009L09Rik | ENSMUSG00000110358 | 1.64 |
| Fam43a | ENSMUSG00000046546 | -1.26 |
| Ip6k3 | ENSMUSG00000024210 | -1.6 |
| Hdac5 | ENSMUSG00000008855 | -1.11 |
| Rbis | ENSMUSG00000078784 | 1.19 |
| Olfr648 | ENSMUSG00000042909 | 2.71 |
| Gpc4 | ENSMUSG00000031119 | 1.41 |
| Pcdhgb1 | ENSMUSG00000103037 | -1.19 |
| Tmem254a | ENSMUSG00000072676 | -1.55 |
| Grik2 | ENSMUSG00000056073 | -1.18 |
| Tmem71 | ENSMUSG00000036944 | -1.64 |
| Slc47a1 | ENSMUSG00000010122 | 1.75 |
| Fam114a1 | ENSMUSG00000029185 | 1.47 |
| Rab38 | ENSMUSG00000030559 | 1.47 |
| Hopx | ENSMUSG00000059325 | 1.25 |
| Txn2 | ENSMUSG00000005354 | 1.26 |
| Ccr2 | ENSMUSG00000049103 | 4.08 |
| Olig3 | ENSMUSG00000045591 | 2.93 |
| Fcgr2b | ENSMUSG00000026656 | 1.71 |
| Zic1 | ENSMUSG00000032368 | 1.38 |
| Chil3 | ENSMUSG00000040809 | 2.86 |
| Olfr1564 | ENSMUSG00000096169 | -1.67 |
| Nefl | ENSMUSG00000022055 | -1.17 |
| Camkk2 | ENSMUSG00000029471 | -1.12 |
| Pdlim1 | ENSMUSG00000055044 | -1.88 |
| Lrtm2 | ENSMUSG00000055003 | 1.18 |
| Hist1h2ap | ENSMUSG00000094777 | -1.87 |
| Dynlt1c | ENSMUSG00000000579 | 1.24 |
| Baiap3 | ENSMUSG00000047507 | -1.24 |
| Prrg4 | ENSMUSG00000027171 | 2.1 |
| Slc6a2 | ENSMUSG00000055368 | -2.14 |
| Iigp1 | ENSMUSG00000054072 | 2.25 |
| Ube2g1 | ENSMUSG00000020794 | 1.18 |
| Zic4 | ENSMUSG00000036972 | 1.7 |
| Cers2 | ENSMUSG00000015714 | 1.18 |
| Slc7a3 | ENSMUSG00000031297 | -1.39 |
| Rpp25 | ENSMUSG00000062309 | -1.43 |
| Cluh | ENSMUSG00000020741 | 1.12 |
| Nyx | ENSMUSG00000051228 | -1.18 |
| Naa10 | ENSMUSG00000031388 | 1.24 |
| Clca1 | ENSMUSG00000028255 | -1.31 |
| Caps2 | ENSMUSG00000035694 | 2.25 |
| Dctn1 | ENSMUSG00000031865 | -1.17 |
| Cyp2g1 | ENSMUSG00000049685 | -4.02 |
| Nckap5 | ENSMUSG00000049690 | -1.18 |
| Rhoj | ENSMUSG00000046768 | 1.31 |
| Dio3 | ENSMUSG00000075707 | 1.59 |
| Slc25a21 | ENSMUSG00000035472 | 2.12 |
| Hmgcr | ENSMUSG00000021670 | -1.11 |
| Cmip | ENSMUSG00000034390 | -1.2 |
| Spon2 | ENSMUSG00000037379 | -1.47 |
| Arg1 | ENSMUSG00000019987 | 1.68 |
| Bace2 | ENSMUSG00000040605 | 1.64 |
| Uqcr11 | ENSMUSG00000020163 | 1.18 |
| Aif1 | ENSMUSG00000024397 | 1.99 |
| Myl9 | ENSMUSG00000067818 | 1.37 |
| Il33 | ENSMUSG00000024810 | 1.28 |
| Itprid2 | ENSMUSG00000027007 | 1.17 |
| Abca9 | ENSMUSG00000041797 | 1.34 |
| Rbfox2 | ENSMUSG00000033565 | -1.11 |
| Metrnl | ENSMUSG00000039208 | 1.91 |
| Sebox | ENSMUSG00000001103 | 1.17 |
| Cldn2 | ENSMUSG00000047230 | 1.77 |
| Tspan4 | ENSMUSG00000025511 | 1.28 |
| Nop53 | ENSMUSG00000041560 | -1.17 |
| Abat | ENSMUSG00000057880 | 1.11 |
| Zfp444 | ENSMUSG00000044876 | -1.17 |
| Atxn10 | ENSMUSG00000016541 | -1.11 |
| Nudt4 | ENSMUSG00000020029 | 1.16 |
| Bcam | ENSMUSG00000002980 | 1.34 |
| Cd36 | ENSMUSG00000002944 | 1.44 |
| Hist1h4n | ENSMUSG00000069305 | -1.36 |
| Bcar3 | ENSMUSG00000028121 | 1.19 |
| Mrpl55 | ENSMUSG00000036860 | 1.27 |
| AL731706.1 | ENSMUSG00000115423 | 1.35 |
| Mpeg1 | ENSMUSG00000046805 | 1.47 |
| Zmat4 | ENSMUSG00000037492 | -1.11 |
| Col23a1 | ENSMUSG00000063564 | 1.17 |
| Lrfn5 | ENSMUSG00000035653 | -1.17 |
| mt-Co1 | ENSMUSG00000064351 | 1.16 |
| Sft2d2 | ENSMUSG00000040848 | 1.17 |
| Abhd18 | ENSMUSG00000037818 | 1.17 |
| Fahd2a | ENSMUSG00000027371 | 1.26 |
| Sorcs2 | ENSMUSG00000029093 | -1.11 |
| Epha6 | ENSMUSG00000055540 | -1.22 |
| Zfp365 | ENSMUSG00000037855 | 1.11 |
| Mycn | ENSMUSG00000037169 | -1.29 |
| Ahdc1 | ENSMUSG00000037692 | -1.17 |
| Cd1d1 | ENSMUSG00000028076 | 1.49 |
| Marc'1 | ENSMUSG00000026621 | -1.77 |
| Ncor2 | ENSMUSG00000029478 | -1.17 |
| Adamts1 | ENSMUSG00000022893 | -1.17 |
| Parn | ENSMUSG00000022685 | 1.17 |
| Ust | ENSMUSG00000047712 | 1.22 |
| Pcdhgb5 | ENSMUSG00000103749 | -1.18 |
| Pm20d1 | ENSMUSG00000042251 | 1.64 |
| H2-T23 | ENSMUSG00000067212 | 1.42 |
| Dohh | ENSMUSG00000078440 | -1.33 |
| Nrxn3 | ENSMUSG00000066392 | -1.16 |
| Cybb | ENSMUSG00000015340 | 1.84 |
| Gm49322 | ENSMUSG00000035370 | -1.24 |
| Snx31 | ENSMUSG00000013611 | 2.34 |
| Gm21992 | ENSMUSG00000096370 | -1.26 |
| Top2a | ENSMUSG00000020914 | -2.59 |
| Efnb2 | ENSMUSG00000001300 | 1.4 |
| Slc6a12 | ENSMUSG00000030109 | 1.34 |
| Mmut | ENSMUSG00000023921 | 1.17 |
| Chdh | ENSMUSG00000015970 | 1.44 |
| Srp14 | ENSMUSG00000009549 | 1.17 |
| Bmp4 | ENSMUSG00000021835 | 1.47 |
| Chia1 | ENSMUSG00000062778 | -2.67 |
| Shisa2 | ENSMUSG00000044461 | 1.24 |
| Lbh | ENSMUSG00000024063 | -1.11 |
| Omd | ENSMUSG00000048368 | 2.77 |
| Anxa7 | ENSMUSG00000021814 | 1.17 |
| Fbn1 | ENSMUSG00000027204 | 1.42 |
| Gpat2 | ENSMUSG00000046338 | 2.17 |
| Shisal1 | ENSMUSG00000062760 | -1.16 |
| Col7a1 | ENSMUSG00000025650 | -1.18 |
| Tbx22 | ENSMUSG00000031241 | 1.95 |
| Elavl2 | ENSMUSG00000008489 | -1.18 |
| Gm14296 | ENSMUSG00000074527 | -1.25 |
| Samd4b | ENSMUSG00000109336 | -1.22 |
| Hist1h2br | ENSMUSG00000069303 | 1.74 |
| Sdc1 | ENSMUSG00000020592 | 1.76 |
| Abhd11 | ENSMUSG00000040532 | 1.2 |
| Pde6d | ENSMUSG00000026239 | -1.21 |
| Aldob | ENSMUSG00000028307 | 1.95 |
| Basp1 | ENSMUSG00000045763 | -1.11 |
| Wfikkn2 | ENSMUSG00000044177 | 1.31 |
| Axl | ENSMUSG00000002602 | 1.23 |
| Spc25 | ENSMUSG00000005233 | -1.16 |
| Pdzd7 | ENSMUSG00000074818 | -1.48 |
| Nat8 | ENSMUSG00000030004 | 2.11 |
| Ckap4 | ENSMUSG00000046841 | -1.17 |
| Nrtn | ENSMUSG00000039481 | -1.78 |
| Gm12184 | ENSMUSG00000078154 | 2.31 |
| Ebf1 | ENSMUSG00000057098 | -1.17 |
| Fscn1 | ENSMUSG00000029581 | -1.17 |
| Hpgd | ENSMUSG00000031613 | 1.23 |
| Hbb-bs | ENSMUSG00000052305 | 1.32 |
| Pcdhga6 | ENSMUSG00000103793 | -1.17 |
| Fgf12 | ENSMUSG00000022523 | -1.11 |
| Pcdhga8 | ENSMUSG00000103897 | -1.17 |
| Abr | ENSMUSG00000017631 | -1.11 |
| She | ENSMUSG00000046280 | -1.57 |
| Med10 | ENSMUSG00000021598 | 1.23 |
| Scrt1 | ENSMUSG00000048385 | -1.27 |
| H4c14 | ENSMUSG00000091405 | -1.24 |
| Apex2 | ENSMUSG00000025269 | -1.16 |
| Foxj1 | ENSMUSG00000034227 | -1.45 |
| Kif5a | ENSMUSG00000074657 | -1.1 |
| Ovca2 | ENSMUSG00000038268 | 1.17 |
| Slc26a6 | ENSMUSG00000023259 | 1.17 |
| Cmbl | ENSMUSG00000022235 | 1.5 |
| Fbrsl1 | ENSMUSG00000043323 | -1.16 |
| Rasef | ENSMUSG00000043003 | 1.33 |
| Nkapl | ENSMUSG00000059395 | -1.52 |
| Cnp | ENSMUSG00000006782 | -1.19 |
| Ptafr | ENSMUSG00000056529 | 2.51 |
| Tnfsf10 | ENSMUSG00000039304 | 1.79 |
| Il1a | ENSMUSG00000027399 | -2.69 |
| Mrpl12 | ENSMUSG00000039640 | 1.37 |
| Zfp366 | ENSMUSG00000050919 | -1.51 |
| Gmpr | ENSMUSG00000000253 | 1.27 |
| Gpr101 | ENSMUSG00000036357 | -2.19 |
| Abhd8 | ENSMUSG00000007950 | -1.16 |
| Abhd17a | ENSMUSG00000003346 | -1.16 |
| Trim30d | ENSMUSG00000057596 | 1.67 |
| Enpep | ENSMUSG00000028024 | 1.43 |
| Chac1 | ENSMUSG00000027313 | -1.49 |
| Ski | ENSMUSG00000029050 | -1.16 |
| Gpr152 | ENSMUSG00000044724 | -1.1 |
| Gsta4 | ENSMUSG00000032348 | 1.24 |
| Nr2f2 | ENSMUSG00000030551 | 1.35 |
| Mrpl21 | ENSMUSG00000024829 | 1.2 |
| P4ha3 | ENSMUSG00000051048 | -2.26 |
| Nectin3 | ENSMUSG00000022656 | 1.23 |
| Ebf3 | ENSMUSG00000010476 | -1.17 |
| Zfp385a | ENSMUSG00000000552 | -1.16 |
| Pcdhb10 | ENSMUSG00000045657 | -1.22 |
| Tubb2b | ENSMUSG00000045136 | -1.23 |
| Kcnj12 | ENSMUSG00000042529 | -1.25 |
| Map4k2 | ENSMUSG00000024948 | 1.11 |
| Setmar | ENSMUSG00000034639 | 1.3 |
| Terb1 | ENSMUSG00000052616 | -1.3 |
| Prdx6 | ENSMUSG00000026701 | 1.15 |
| Fam122a | ENSMUSG00000074922 | -1.44 |
| Jph4 | ENSMUSG00000022208 | -1.26 |
| Tubb4a | ENSMUSG00000062591 | -1.16 |
| Rpl39 | ENSMUSG00000079641 | 1.16 |
| Bmp7 | ENSMUSG00000008999 | 1.3 |
| Stxbp2 | ENSMUSG00000004626 | 1.21 |
| Fuca2 | ENSMUSG00000019810 | 1.16 |
| Ech1 | ENSMUSG00000053898 | 1.16 |
| Gbp4 | ENSMUSG00000079363 | 1.78 |
| Hbb-bt | ENSMUSG00000073940 | 1.41 |
| Prrx1 | ENSMUSG00000026586 | 1.61 |
| Acyp2 | ENSMUSG00000060923 | 1.28 |
| Ptn | ENSMUSG00000029838 | 1.16 |
| Sipa1l3 | ENSMUSG00000030583 | -1.16 |
| Timm17a | ENSMUSG00000062580 | -1.11 |
| Wdr72 | ENSMUSG00000044976 | 1.7 |
| Clybl | ENSMUSG00000025545 | 1.37 |
| Fbln1 | ENSMUSG00000006369 | 1.68 |
| Trim30a | ENSMUSG00000030921 | 1.54 |
| Gucy2c | ENSMUSG00000042638 | 2.31 |
| Zbtb7a | ENSMUSG00000035011 | -1.21 |
| Creld1 | ENSMUSG00000030284 | -1.17 |
| Ddx60 | ENSMUSG00000037921 | 1.29 |
| C1qtnf4 | ENSMUSG00000040794 | -1.44 |
| Sptbn4 | ENSMUSG00000011751 | -1.2 |
| Lrp1b | ENSMUSG00000049252 | -1.15 |
| Ccdc136 | ENSMUSG00000029769 | -1.24 |
| Rgs7bp | ENSMUSG00000021719 | -1.1 |
| Atf5 | ENSMUSG00000038539 | -1.31 |
| Bsn | ENSMUSG00000032589 | -1.15 |
| Pkp2 | ENSMUSG00000041957 | 1.17 |
| Oga | ENSMUSG00000025220 | -1.1 |
| Slc9a3 | ENSMUSG00000036123 | 1.84 |
| Nos2 | ENSMUSG00000020826 | 2.59 |
| Spint1 | ENSMUSG00000027315 | -1.56 |
| Trim66 | ENSMUSG00000031026 | -1.21 |
| Clvs2 | ENSMUSG00000019785 | -1.33 |
| Mthfd2 | ENSMUSG00000005667 | -1.26 |
| Foxc1 | ENSMUSG00000050295 | 1.6 |
| Marcksl1 | ENSMUSG00000047945 | -1.17 |
| Col25a1 | ENSMUSG00000058897 | 1.16 |
| Tead4 | ENSMUSG00000030353 | 2.91 |
| Pnpla3 | ENSMUSG00000041653 | -1.16 |
|  |  |  |
